# Distributions of Transposable Elements Reveal Hazardous Zones in Mammalian Introns

**DOI:** 10.1371/journal.pcbi.1002046

**Published:** 2011-05-05

**Authors:** Ying Zhang, Mark T. Romanish, Dixie L. Mager

**Affiliations:** 1 Terry Fox Laboratory, British Columbia Cancer Agency, Vancouver, British Columbia, Canada; 2 Department of Medical Genetics, University of British Columbia, Vancouver, British Columbia, Canada; Jefferson Medical College/Thomas Jefferson University, United States of America

## Abstract

Comprising nearly half of the human and mouse genomes, transposable elements (TEs) are found within most genes. Although the vast majority of TEs in introns are fixed in the species and presumably exert no significant effects on the enclosing gene, some markedly perturb transcription and result in disease or a mutated phenotype. Factors determining the likelihood that an intronic TE will affect transcription are not clear. In this study, we examined intronic TE distributions in both human and mouse and found several factors that likely contribute to whether a particular TE can influence gene transcription. Specifically, we observed that TEs near exons are greatly underrepresented compared to random distributions, but the size of these “underrepresentation zones” differs between TE classes. Compared to elsewhere in introns, TEs within these zones are shorter on average and show stronger orientation biases. Moreover, TEs in extremely close proximity (<20 bp) to exons show a strong bias to be near splice-donor sites. Interestingly, disease-causing intronic TE insertions show the opposite distributional trends, and by examining expressed sequence tag (EST) databases, we found that the proportion of TEs contributing to chimeric TE-gene transcripts is significantly higher within their underrepresentation zones. In addition, an analysis of predicted splice sites within human long terminal repeat (LTR) elements showed a significantly lower total number and weaker strength for intronic LTRs near exons. Based on these factors, we selectively examined a list of polymorphic mouse LTR elements in introns and showed clear evidence of transcriptional disruption by LTR element insertions in the *Trpc6* and *Kcnh6* genes. Taken together, these studies lend insight into the potential selective forces that have shaped intronic TE distributions and enable identification of TEs most likely to exert transcriptional effects on genes.

## Author Summary

Sequences derived from transposable elements (TEs) are major constituents of mammalian genomes and are found within introns of most genes. While nearly all TEs within introns appear harmless, some *de novo* intronic TE insertions do disrupt gene transcription and splicing and cause disease. It is unclear why some intronic TEs perturb gene transcription whereas most do not. Here, we examined intronic TE distributions in both human and mouse genes to gain insight into which TEs may be more likely to affect transcription. We found evidence that TEs near exons are likely subject to strong negative selection but the size of the region under selection or “underrepresentation zone” differs for different TE classes. Strikingly, all reported human disease-causing intronic TE insertions fall within these underrepresentation zones, and the proportion of TEs contributing to chimeric TE-gene transcripts is significantly higher when TEs are located in these zones. We also examined insertionally polymorphic mouse TEs located within underrepresentation zones and found evidence of transcriptional disruption in two genes. Given the growing appreciation for ongoing activity of TEs in human, our results should be of value in prioritizing insertionally polymorphic TEs for study of their potential contributions to gene expression differences and phenotypic variability.

## Introduction

Transposable Elements (TEs) are major factors that have shaped the landscape of the mammalian genome through evolution. Most TEs in mammals are inactive remnants of ancient TE insertions, buried in the host genome for millions of years. In rodents and primates, TEs comprise 38–45% of the genome [1], [2], and about 90% of all human RefSeq genes contain TEs in their introns. These TEs can be divided into four major classes: long interspersed elements (LINEs), short interspersed elements (SINEs), long terminal repeat (LTR) retroelements (including endogenous retroviruses (ERVs)), and DNA transposons [3]. The first three classes are retrotransposons, which utilize an RNA intermediate during their retrotransposition process and account for most TEs in mammalian genomes. On the other hand, DNA transposons move directly to new genomic loci without being reverse-transcribed. Although most mammalian TEs are neutral components of the genome with no significant biological effects [4], [5], some elements do impact the cell/organism by acting as insertional mutagens, inducing DNA rearrangements, assuming cellular functions and altering gene regulation [4], [6], [7], [8].

Biologically significant TEs are usually discovered and studied on a case-by-case basis, although bioinformatics approaches have also been used to identify potentially functional TEs. Genomic comparisons between species have identified deeply conserved TEs that function as regulatory elements [9], . TEs that serve as alternative exons, promoters or polyadenylation signals are also straightforward to detect by looking for chimeric transcripts between the TE and neighboring genes [11], [12], [13], [14]. Global TE distribution patterns in mammalian genomes have been intensely studied in the past decade, and such analyses have provided insight into the selective forces that influence fixation probabilities of TE insertions. For example, some studies have evaluated the relationships between TE distributions and imprinted genes [15], and gene expression patterns [16], [17], [18]. TE-free regions have also been used as markers to identify potentially critical regulatory regions [19], [20]. Moreover, it is clear that LTR elements and LINEs are more prevalent in intergenic regions compared to gene introns, and most of those that do reside in gene introns are in the antisense orientation with respect to the enclosing genes [3], [21]. This pattern reflects stronger selection against sense-oriented elements, likely due to the greater chance that such elements will disrupt gene transcript processing [22].

While cases have been reported of influential TEs far from genes, those elements near or within genes likely have a greater potential of affecting gene expression. However, our current knowledge of the distribution of TEs *within* gene introns is very limited, and it remains unclear why some intronic TEs perturb gene transcription while most do not. To fully understand their biological effects, it would be useful to determine which intronic TEs are most likely to affect gene expression, so they can be prioritized for functional analyses. With a growing appreciation for SINE and LINE insertional polymorphisms in human [23], [24], [25], [26], [27], [28], such predictions would be particularly helpful in identifying polymorphic TE insertions with the greatest probability of affecting gene transcription and, therefore, possibly contributing to phenotypic variability or disease susceptibility in humans. In this study, we conducted a set of bioinformatics analyses of TE distribution patterns within human and mouse genes and revealed TE underrepresentation zones and distributional biases in gene introns. TEs that do occur within the underrepresentation zones are more likely to be involved in aberrant gene splicing and known cases of intronic disease-causing TE insertions are primarily located within these zones, strongly suggesting that TEs in these locations are more likely to be harmful and be selected against. The results of our study reveal a distinct tendency for TEs to affect gene transcription when poised near exons, and point to their continued role in catalyzing genome evolution.

## Results/Discussion

### Intronic regions near exon boundaries are depleted of TE insertions

According to our genomic survey, 85–90% of mouse and human protein coding genes contain TE sequences in their introns. In a recent study of the relationship between Alu SINEs and alternative splicing, Lev-Maor et al. reported a drop of Alu density within 150 bp from intron boundaries [29]. Based on this observation and the fact that most intronic splice signals are located at the 5′- and 3′-end of introns [30], we hypothesized that *de novo* intronic TE insertions near exons are more likely to be mutagenic, and consequently, that the frequency of TEs would be significantly lower than expected in general near intron ends.

**Figure 1:**
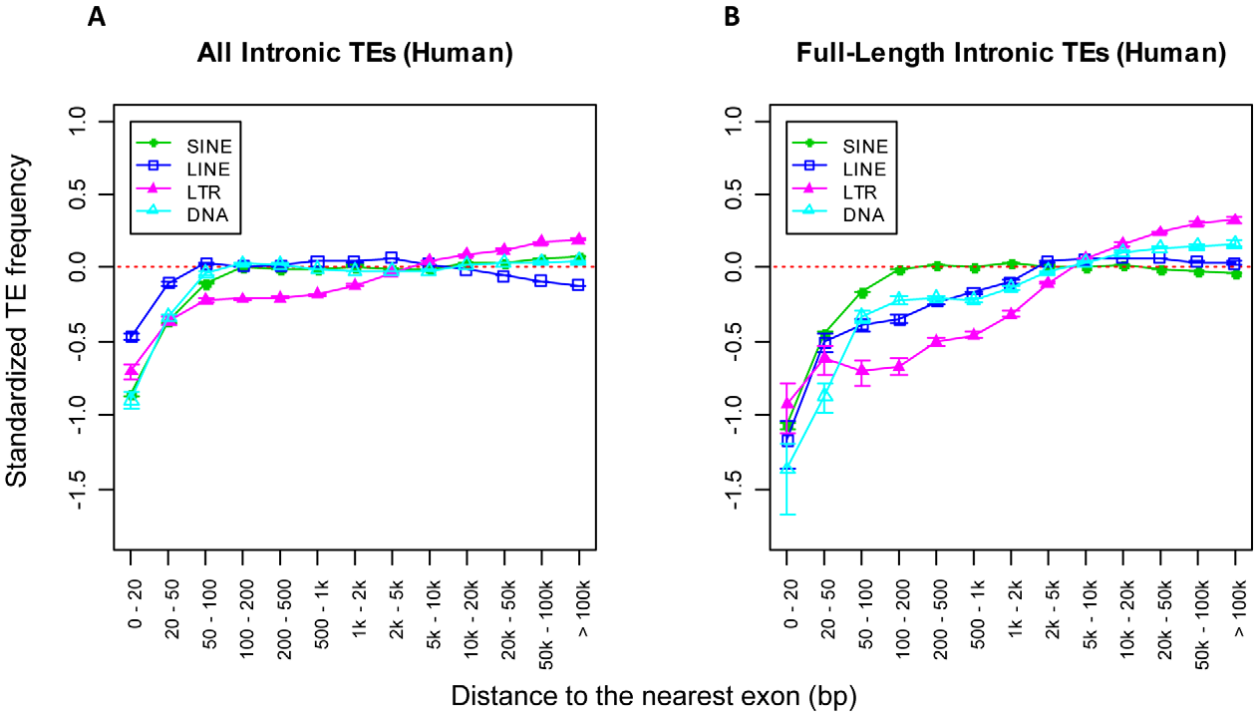
Intronic distributions of the four major TE classes in human (normalized). The distributions of all (A) and full-length (B) intronic TEs in human are shown separately. The sizes of the U-zone observed for each TE class are specified in Table 1. In both A and B, the x-axis shows a series of predefined intronic regions based on the distance from a TE to the nearest exon. The y-axis represents the standardized frequency of TEs at each intronic region and is normalized by G/C content for each TE class. The red dotted line indicates the expected distribution of TEs based on random computational simulations. Error bars are standard errors derived from the total number of corresponding TEs (sample size) in each bin.

To analyze the distributions of various TE classes within introns, we first conducted computer simulations to determine theoretical TE distribution patterns (see Materials and Methods). Then we determined the actual distribution pattern of intronic TEs according to their distance to the nearest exon. To alleviate our concern about the potential effect of “distance shifting”- a hypothesized result of later TE insertions or other rearrangements occurring between a specific TE and its nearest exon, we also analyzed the distribution of the youngest 20% of intronic TEs. However, we observed only minor differences compared to all intronic TEs in the genome (data not shown). To clearly show the difference between simulated and actual TE distributions at each predefined position in introns, we calculated the ‘standardized frequency’ of observed TEs (see Materials and Methods). Briefly, the level of TE representation at each predefined intronic interval is determined from the difference between the actual TE distribution in the genome (observed) and the computer simulation of random TE insertions (expected). When this value is positive, it reflects an overrepresentation of a given TE class within the corresponding intronic region; however, when negative it indicates underrepresentation. As expected, we found that all four major TE classes are highly underrepresented near intron boundaries in both human (Figure 1A in Text S1) and mouse (data not shown). We next applied the same distribution analysis for only full-length or near full-length TE sequences (see Table 1 for “full-length” definitions). Again, as shown in Figure 1B in Text S1 for human, full-length TEs were highly underrepresented when close to exons, but most TE classes except SINEs showed larger underrepresentation zones (hereafter shortened to U-zone) compared with the all-TE distributions.

**Table 1:** Intronic underrepresentation zones by TE class.

TE Class	Human U-zone for All (bp)^a^	Mouse U-zone for All (bp)^a^	Human U-zone for FL (bp)^b^	Mouse U-zone for FL (bp)^b^	Human cutoff size of FL (bp)^c^	Mouse cutoff size of FL (bp)^c^
SINE	100	100	100	100	>250	>100
LINE	50	100	2000	2000	>5000	>5000
LTR	2000	1000	5000	2000	>5000	>5000
DNA	50	100	2000	2000	>1000	>1000

The distributions of TEs were normalized by the overall G/C content preference of each TE class.

a Underrepresentation zone based on distribution of all elements of each TE class.

b Underrepresentation zone based on distribution of only ‘near full-length’ (FL) elements.

c The cutoff size of full-length elements for each TE class was determined as slightly shorter than the average full-length elements as described in [1] and [2].

**Figure 2:**
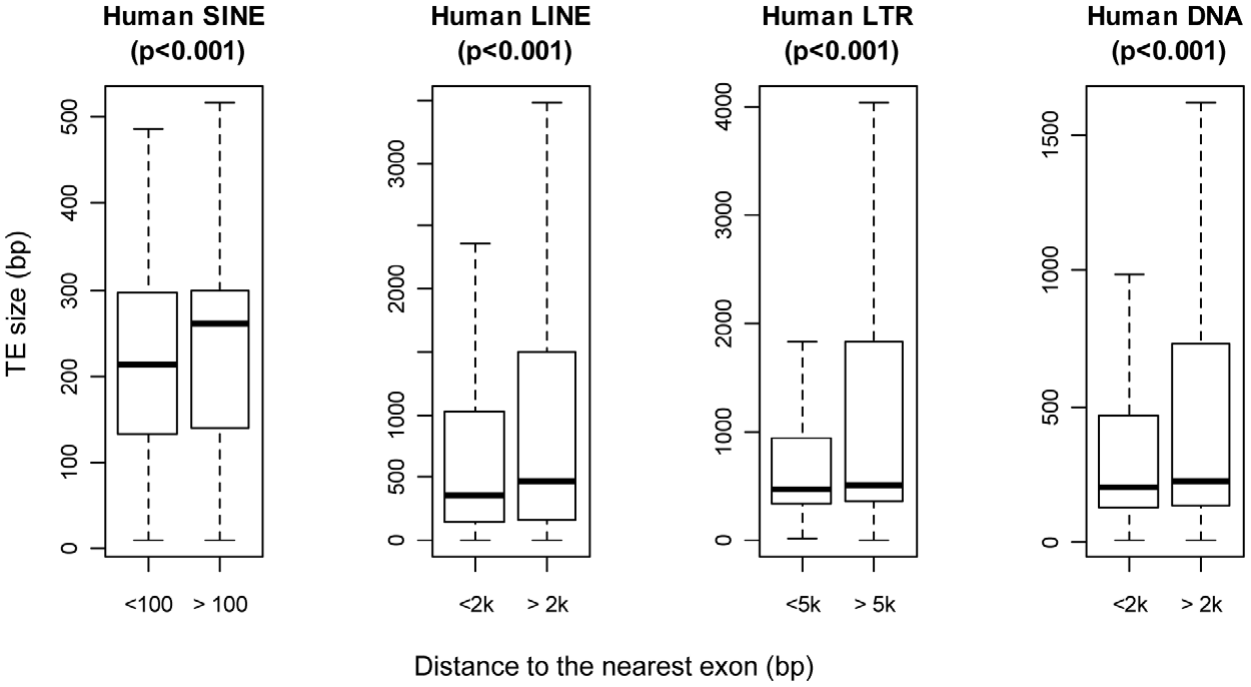
Average size of human TEs within and outside the U-zone. Each TE class is divided into two groups as shown on the x-axis: one group for elements located within the corresponding U-zone of full-length TEs and another group for those beyond. The average size of each TE group is indicated as the horizontal bar within each box, which represents the central 50% of data points of the group. Outliers beyond the 1.5× IQR (interquartile range) whiskers are not shown. P-values shown on top of each boxplot are based on the two-sample Wilcoxon test.

We also noticed that intronic regions more than 20 kb from exons showed a significant underrepresentation of SINEs compared to random simulations. Unlike patterns close to exons, intronic TE distributions greater than 20 kb from exons are less likely due to purifying selection so we searched for other explanations. SINE elements are more abundant in G/C-rich regions [1], [21] and, since large introns resemble intergenic regions in terms of G/C content (which is generally A/T rich) [31], we postulated that the drop of SINE frequency compared to random simulations in deep intronic regions was an effect of local G/C content. To determine if this was the case, we normalized our random simulations with the local G/C content as described in Materials and Methods. Indeed, after applying such normalization, the underrepresentation of SINEs in deep intronic regions greatly flattened out, while the sizes of the U-zones near exons were not affected. Hence all our subsequent analyses employed this normalization. Figure 1 shows the normalized plots for all human TEs (Figure 1A) and full length TEs (Figure 1B), and these plots are very similar for mouse TEs (Figure 2 in Text S1). Interestingly, the sizes of the U-zones near intron boundaries are different between TE classes (Table 1).

Original insertion site preferences, natural selection and genetic drift could all contribute to global TE distributions. While determining the initial integration site preference of TEs is difficult if not impossible (especially for ancient families), a limited number of *de novo* TE integration studies showed that TEs in today's human genome are distributed very differently from their initial target site preferences [32], [33]. Indeed, since 99% of TEs in the human genome and 93% in the mouse genome have been fixed for more than 25 million years [1], it is reasonable that their current distributions will bear little resemblance to any original insertion site preferences but will primarily be the result of selection and genetic drift. Therefore, the TE U-zones identified here most likely result from purifying selection, rather than original avoidance of these regions during the integration process.

### TEs within their U-Zones are shorter

The larger U-zones for full length TEs (compare Figures 1A and B) suggests that purifying selection acts at much greater distances on full-length elements than on their partly deleted counterparts. This effect is not observed for SINEs but these elements have a much shorter full-length size (∼300 bp for human Alus) [1], [8], will generally carry fewer cryptic transcriptional regulatory signals and are less harmful to the enclosing genes than other TEs [34]. For the above reasons, full-length SINE elements may be better tolerated at a closer distance to exons.

**Figure 3:**
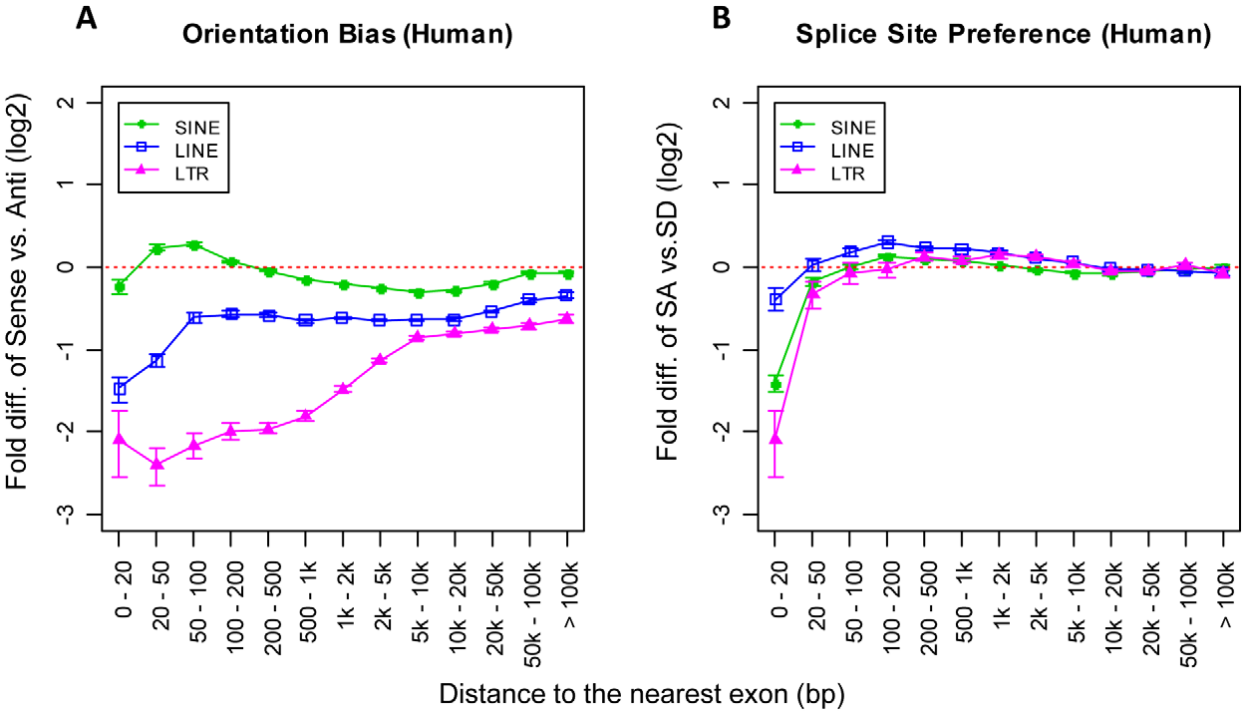
Distributional biases of full-length human intronic TEs. A) Orientation bias of full-length intronic TEs. The y-axis shows the logarithmic fold-difference of TE frequency between sense and antisense oriented full-length TEs. B) Splice site bias of full-length intronic TEs. The y-axis shows the logarithmic fold-difference of full-length TE frequency between TEs close to the SA site and TEs close to the SD site. The x-axis shows a series of predefined intronic regions based on the distance from a TE to the nearest exon. Error bars are standard errors derived from the total number of corresponding TEs (sample size) in each bin.

We next compared the average length of intronic TEs within and outside their full-length U-zones and found a significant difference for all TE classes in both species (Figure 2 for human; Figure 3 in Text S1 for mouse). In fact, most elements within their respective U-zones are truncated, while a greater portion of TEs beyond such zones are full-size elements, resulting in a much bigger size variance (see the difference between upper whiskers in Figure 2 for human and also Figure 3 in Text S1 for mouse). Therefore, the length of individual TEs is an important aspect dictating their genomic distributions, indicating that larger elements are more likely to be genotoxic when positioned near exons. These results also support previous work regarding L1 LINEs, indicating that, compared to shorter elements, full length L1s have more potentially disruptive splice and polyadenylation signals [35], have greater effects on expression of enclosing genes [36] and have a greater fitness cost [37].

### TEs near exons exhibit strong orientation and splice-site bias

**Figure 4:**
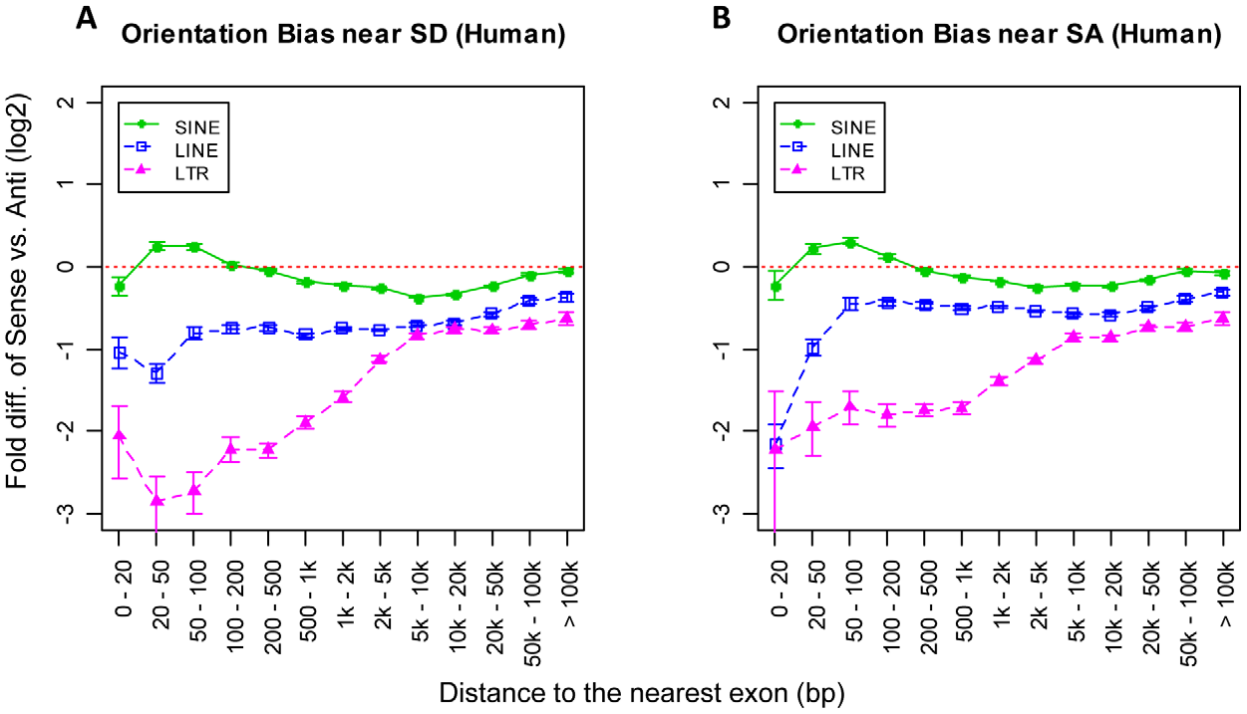
Orientation bias of human full-length intronic TEs based on their proximity to different types of splice sites. Orientation bias of full-length TEs near SD sites (A) and SA sites (B). The x-axis shows a series of predefined intronic regions based on the distance from a TE to the nearest exon. The y-axis shows the logarithmic fold-difference of TE frequency between sense and antisense oriented TEs. Error bars are standard errors derived from the total number of corresponding TEs (sample size) in each bin.

We next examined the distribution of intronic TEs in the sense orientation versus those in antisense with respect to the enclosing genes (see Figure 3A for human and Figure 4A in Text S1 for mouse). Since DNA transposons only comprise about 3% of both the human and the mouse genomes and almost all of them are ancient elements without evidence of any transposition activity during the past 50 Myr (million years) [1], [2], we excluded them from the following analyses to avoid uncertainties introduced by their relatively small numbers. While previous studies have found an overall antisense orientation bias in genes (particularly for LTR elements and LINEs) [21], [22], we show here the existence of a much stronger bias in antisense for both LINEs and LTR elements near exons. The excess of antisense TEs compared with sense elements near intron boundaries is probably the result of purifying selection, like the genome-wide orientation bias of TEs in genes. This indicates in general that sense-oriented TEs near splice sites have a higher probability to influence normal gene transcription and are potentially more harmful to the host gene. Interestingly, for SINEs we observed the same strong antisense bias in the mouse (Figure 4A in Text S1), but in the human genome we observed a *sense* orientation bias instead of antisense for SINEs at a close distance of 20–200 bp from exons (Figure 3A). These data are consistent with the Alu SINE study of Lev-Maor et al. [29], in which the authors also observed more sense-oriented Alu elements near intron termini. Since Alus account for two-thirds of human SINE elements and many antisense Alus possess a strong cryptic SA signal [13], selection against antisense-oriented elements may explain the unusual underrepresentation of antisense oriented SINEs near splice sites in humans.

Furthermore, we also looked for evidence of any distributional bias of intronic TEs in terms of their proximity to either splice donor sites (SDs) or splice acceptor sites (SAs). We found the total numbers of elements near SA sites are much lower than SD sites for all three retrotransposon classes examined (see Figure 3B for human and Figure 4B in Text S1 for mouse). Since the core intronic splice signals at SD sites usually only consist of about 6 bp of terminal intron sequence compared with 20–50 bp at SA sites [30], selection against physical disruption of critical splice motifs likely underlies this TE underrepresentation near SA sites.

**Figure 5:**
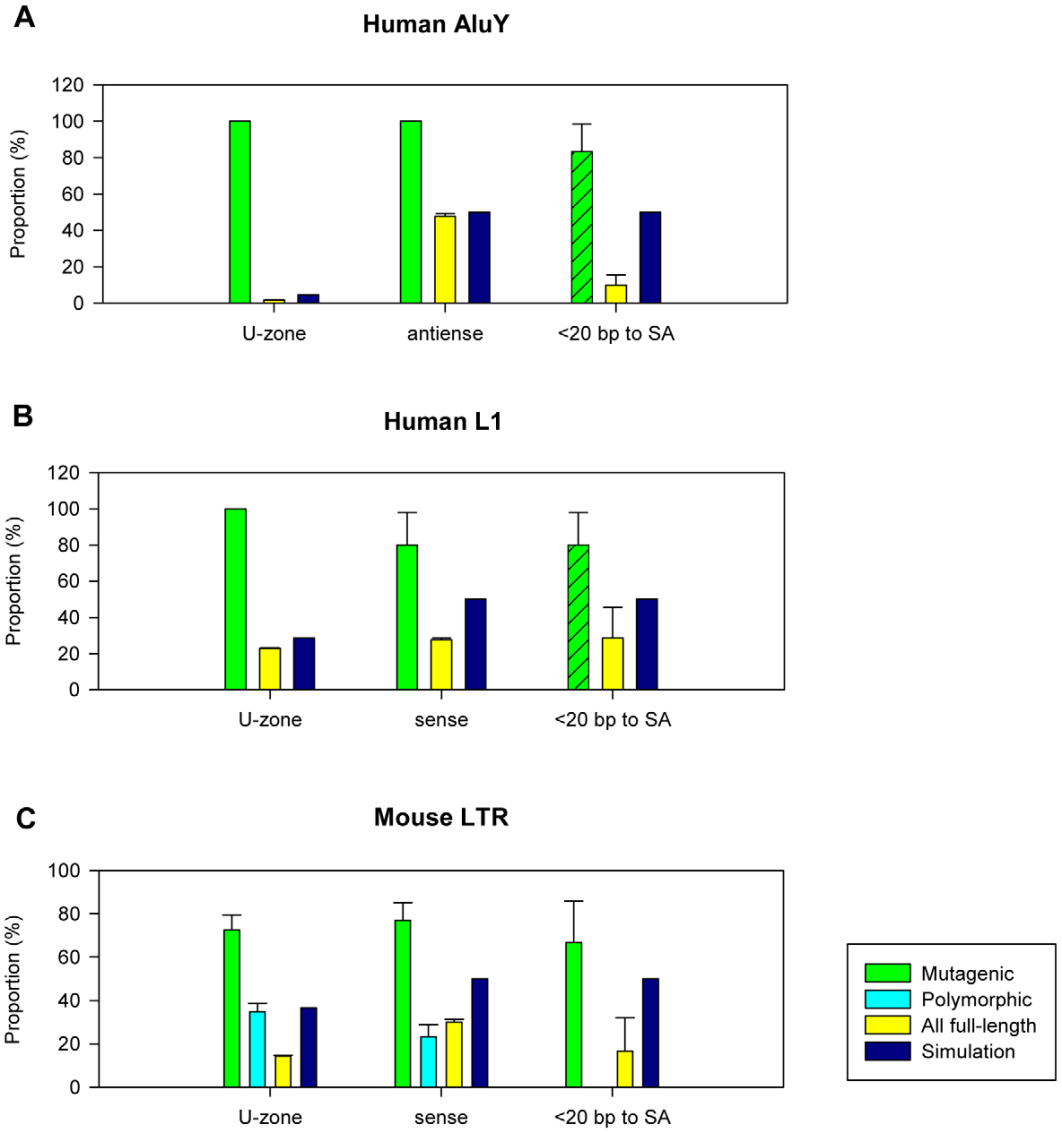
Comparisons of TE frequency within the U-zone. Three TE classes were examined and results were plotted in panel A, B, and C for the human Alu, human L1, and mouse LTR elements, respectively. In each plot, three groups of comparisons are shown: ‘U-zone’ stands for TE insertions within the U-zone; ‘antisense’ for human Alu or ‘sense’ for human L1 and mouse LTR indicates TEs within the U-zone in the corresponding orientation with respect to the enclosing gene; ‘<20 bp to SA’ indicates TE insertions within 20 bp of SA sites with an exception for human Alu and L1 mutagenic TEs (marked by shading), for which all cases were included due to limited total numbers. The y-axis shows the percentage of TEs that belong to the corresponding groups. Bars in each group represent mutagenic TE insertions (green), polymorphic TE insertions (light blue), all full-length TE insertions in the reference genome (yellow), and computational simulation as a random control (dark blue). Error bars represent standard errors derived from the total number of cases (sample size) for each category.

Theoretically, harmful antisense transcripts of protein-coding exons may be generated by read-through transcription of antisense TEs near SD sites. If such antisense transcripts have significant detrimental effects, then one might expect a larger proportion of TEs near SD sites to be in sense rather than in antisense due to purifying selection. However, as shown in Figure 4A (human) and Figure 5A in Text S1 (mouse), such predicted bias of sense orientated TEs near SD sites was not found except for human SINEs, which is likely explained by the fact mentioned previously that antisense Alus possess cryptic SA signals. In fact, for other TE classes we observed more SD-associated elements oriented in antisense, probably indicating that antisense transcription is effectively silenced or not a general problem, and that sense oriented TE insertions are more detrimental. The same analysis of TEs near SA sites revealed similar orientation bias patterns as for TEs near SD sites.

### A high fraction of known mutagenic intronic TEs reside within U-zones

If the reduced frequency of TEs near intron boundaries reflects the force of selection against harmful insertions, one would predict that a higher fraction of mutagenic TEs in gene introns would be located within these TE underrepresentation zones. To evaluate this prediction, we compiled information on documented intronic mutagenic TE insertions and examined their integration sites in introns.

Based on the TE activity and data availability, we focused on the following three TE families in our analyses: human Alu (SINE), human L1 (LINE) and mouse LTR elements. First, as the most abundant TE family, Alus have successfully propagated in the human genome and reached a total number of over one million copies [1]. Even today, some of these elements are still active, generating new insertions and causing mutations linked to diseases [8], [38], [39]. Based on the information provided by the dbRIP database (http://dbrip.brocku.ca/) [27], we found six *de novo* Alu insertions associated with human diseases within introns, all of which belong to the AluY subfamily (the youngest subfamily of Alu) and cause splice defects of the enclosing gene (Table 1 in Text S2). Second, *de novo* disease-causing insertions of L1, the active LINE family in humans, have also been reported [5], [40], [41], [42]. These elements play important roles in human retrotransposon-mediated pathogenesis because not only do they encode reverse-transcriptase (RT) and other proteins required for their own retrotransposition, but also for mobilizing Alus [43]. In this study, our search of the dbRIP database identified a total of five intronic L1s associated with human diseases (Table 2 in Text S2), all of which cause transcriptional disruptions. Last, since no mutagenic LTR insertions and only a few insertionally polymorphic ERVs or LTRs have been reported in human [4], [6], [44], we turned to the mouse genome, where ERVs/LTR elements cause ∼10% of germline mutations, many of which have been well studied [7]. In total we collected 40 cases of mutagenic LTR elements in mice: 15 from the Intracisternal A Particle (IAP) family, 18 from the Early Transposon/Mouse Type D retrovirus (ETn/MusD) family, and seven from other LTR elements or ERVs. Again, all these ERV-induced intronic mutations in mice are due to transcriptional disruptions on the enclosing gene (Table 3 in Text S2).

For the three TE families listed above, we compared the intronic distribution of mutagenic elements with all full-length counterparts in the reference genomes and found highly consistent results (Figure 5 and Table 2). As shown in Figure 5A, all six mutagenic Alu insertions are within the U-zone of SINEs (i.e. <100 bp from the nearest exon), and all are oriented antisense with respect to the enclosing gene. Moreover, five out of the six cases are near SA sites. In comparison, only 1.83% of all full-length AluYs in the reference human genome are located within the 100 bp U-zone - strikingly lower than the mutagenic elements and also more than two-fold lower than that expected by chance (p<2.2e-16; one-sample proportion test). For all full-length AluYs within the U-zone we observed 47.7% elements in antisense, slightly lower than the random level (50%) but much lower than mutagenic insertions. Since intronic TEs show their strongest splice site bias when they are in extreme close proximity to an exon (Figure 3B), we examined full-length intronic AluYs located less than 20 bp from exons and observed only 10% of such elements near SA sites. Although we cannot directly compare this result to the case of mutagenic Alus due to their insufficient number within 20 bp to exons, the fact that five out of six mutagenic Alus are near SAs is noteworthy.

**Table 2:** Intronic distributional biases of mutagenic, polymorphic, and all full-length TEs.

TE Type		Total TE cases	TEs in U-zone/total TEs	Sense TEs/TEs in U-zone	TEs near SA/TEs ≤20 bp to exon
Human Alu	Mutagenic^a^	6	6/6 (100%)	6/6 (100%)	5/6* (83.3%)
	Full-length^b^	54136	989/54136 (1.8%)	472/989 (47.7%)	3/30 (10%)
	Expected^c^		4.5%	50%	50%
Human L1	Mutagenic	5	5/5 (100%)	4/5 (80%)	4/5 (80%)*
	Full-length	10134	2328/10134 (23.0%)	644/2328 (27.7%)	2/7 (28.6%)
	Expected		28.6%	50%	50%
Mouse LTR	Mutagenic	40	29/40 (72.5%)	20/26 (76.9%)	4/6 (66.7%)
	Polymorphic^d^	161	56/161 (34.8%)	13/56 (23.2%)	0/0
	Full-length	10150	1447/10150 (14.3%)	435/1447 (30.1%)	1/6 (16.7%)
	Expected		36.6%	50%	50%

*Due to the limited number of cases, all human Alu and L1 mutagenic insertions are included rather than only using elements within 20 bp to SAs.

a Mutagenic insertions documented in the literature.

b All full-length TEs (see cut-off size of full-length TEs in Table 1) in the reference human/mouse genome.

c Based on random computational simulation.

d Polymorphic ERV insertions present in the B6 mouse reference genome and at least one other mouse strain.

Similarly, Figure 5B shows that all five mutagenic L1 elements are within the U-zone for full-length LINEs (i.e. <2 kb from the nearest exon). Among them, four are sense-oriented and four are near SA sites. In contrast, only 23.0% of full-length intronic L1s in the reference genome are within the U-zone, which is significantly lower than both the mutagenic L1s and our random simulation (p<0.0004 and p<2.2e-16, respectively; two-/one-sample proportion test). Of those elements within the U-zone, only 27.7% are in sense, again significantly lower than both mutagenic insertions and the simulation (p<0.035 and p<2.2e-16, respectively; two-/one-sample proportion test). Although the number of full-length L1s in the reference genome within 20 bp to exons is very limited, among a total of seven cases only two were found near SA sites.

We also examined the same parameters for mouse LTR elements (Figure 5C and Table 2). As we expected, a high fraction of these mutagenic insertions (72.5%) are within the U-zone of full-length mouse LTR elements (i.e. <2 kb from the nearest exon). More remarkably, all 15 mutagenic insertions from the IAP family were within the 2 kb U-zone. Since the orientation information of some mutagenic LTR elements within the U-zone was not indicated in their original reports, we checked the remaining 26 cases and found 20 (76.9%) were oriented in sense. Among these mutagenic insertions in mice, five are located within 20 bp of exons, with three of them near SA sites (60%). However, the situation is completely different for all full-length LTR elements in the sequenced mouse genome (strain C57BL/6J, or B6). In contrast to mutagenic insertions, only 14.3% of full-length LTR elements in the reference genome were located within the 2 kb U-zone (p<2.2e-16; two-sample proportion test), and of these elements only 30.1% are in the sense orientation (p<2.65e-09; two-sample proportion test). At a distance less than 20 bp to exons, we found six full-length LTR elements in the B6 reference genome but only one of them is near the SA site (16.7%).

In summary, the above analyses of mutagenic versus all full-length elements for the three retrotransposon families consistently showed an overrepresentation of mutagenic TEs within their respective U-zones but an underrepresentation of all full-length elements within the same regions. Moreover, apparent differences in orientation and splice-site biases were also observed between mutagenic TEs and all full-length elements in the reference genomes. These observations strongly suggest that intronic TE insertions within the U-zone have a much higher potential to be deleterious to the enclosing gene, particularly when oriented in antisense for human SINEs and in sense for LINEs and LTR elements. When intronic TE insertions are in extreme proximity (e.g. <20 bp) to an SA site, they are very likely to be harmful and may cause functional abnormality of the enclosing gene.

### Polymorphic LTR elements in mice show an intermediate distribution pattern

We next extended our analyses to polymorphic AluY and L1 insertions not associated with any disease based on the dbRIP data. These elements are considered as relatively young since they are not fixed in humans. If, indeed, selection is still working upon these TEs, one might see an intermediate distribution pattern between that of mutagenic and all elements. However, for both polymorphic AluYs and L1s we observed no significant differences from all full-length elements in the reference human genome (data not shown). While the limited total number of polymorphic insertions documented in dbRIP may partially account for this result, it is very likely that the distribution of these polymorphic TEs has already been shaped by selection.

However, for the youngest insertionally polymorphic mouse LTR elements, we have previously shown that they do have a distinct prevalence in introns and orientation bias compared with older elements [45]. This suggests that some of these insertions are detrimental but have not been eliminated due to the artificial breeding environment of inbred strains [2], [7], [45]. Indeed, some known detrimental LTR insertions have even become fixed in one or a few mouse strains [46], [47]. We therefore analyzed a list of polymorphic LTR insertions in four mouse strains from our previous study [45], in which we had detected different distributions between polymorphic and common LTR elements. Here we used polymorphic IAP and ETn/MusD elements that are present in only one of the four analyzed mouse strains (presumed to be the youngest elements) and found that 34.8% of intronic insertions were within the 2 kb U-zone (Figure 5C and Table 2), a fraction very close to the simulated prediction of a random distribution but significantly higher than all full-length LTR elements in the mouse reference genome (14.3%; p<5.58e-13; two-sample proportion test) and lower than the mutagenic insertions (72.5%; p<9.79e-05; two-sample proportion test). Moreover, we observed 23.2% of polymorphic LTRs in the U-zone as sense-oriented, which shows no statistical difference from that of all LTRs but is highly significantly lower than the mutagenic cases (p<6.26e-07; two-sample proportion test). Since our list of polymorphic LTR insertions in mice does not contain any intronic insertions within 20 bp of an exon, we could not perform the analysis of splice site proximity bias. Nonetheless, the above observation of an intermediate distribution pattern of polymorphic insertions between mutagenic and all full-length TEs in the reference genome demonstrates that, indeed, purifying selection is the most likely underlying force shaping the observed intronic TE distribution patterns, and evidence suggests that such a process is ongoing.

### Chimeric transcripts and cryptic splice signals differ within and outside the U-zone

**Figure 6:**
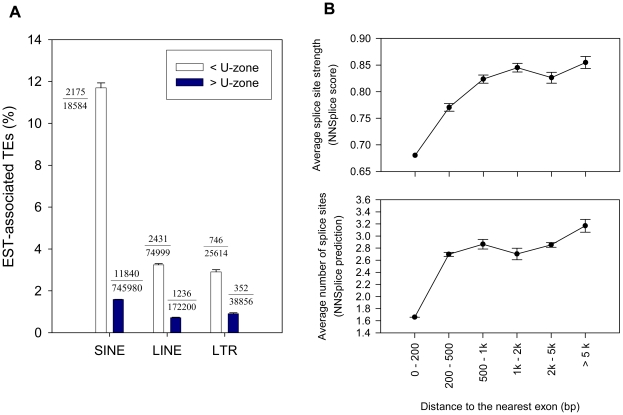
Chimeric transcripts and cryptic splice signals of TEs within and outside the U-zone. A) EST-associated human intronic TEs within and outside the U-zone. Each TE class is shown as a group on the x-axis. The y-axis shows the percentage of intronic TEs that contribute to chimeric ESTs with the enclosing gene. The white/dark bar represents all TEs of each TE class within/outside the U-zone, respectively. The fraction numbers beside each bar indicates the total number of TEs in each category (denominator) and the number of cases involved in chimeric ESTs (numerator). Error bars represent standard errors derived from the total number of cases (sample size) for each category. B) Predicted number and strength of cryptic splice sites in human LTRs. The top panel gives the average strength of predicted splice sites within sampled LTR sequences within each bin (the y-axis) based on the distance from the LTR to its nearest intron boundary (the x-axis). The bottom panel shows the same but for the average total number of predicted slice sites.

If TEs within their respective U-zones are more likely to be harmful by causing splicing abnormalities, one can make two predictions. One prediction is that TEs located in the U-zones would be associated with chimeric TE-gene transcripts more often than TEs located elsewhere in introns. To test this prediction, we downloaded and analyzed the human expressed sequence tag (EST) data from the UCSC Genome Browser (http://genome.ucsc.edu), in which only spliced transcripts were included. We then screened for all spliced ESTs overlapping with intronic TEs (i.e. chimeric ESTs). As shown in Figure 6A, we observed that 11.7% of human SINE elements within the 100 bp U-zone are associated with chimeric ESTs. In contrast, this ratio is only 1.6% for SINE elements outside the U-zone. Similarly, for human LINEs in their 2 kb U-zone, we found 4.6% of them associated with chimeric ESTs, while outside the U-zone the ratio significantly drops to 0.7%. Lastly, we identified 2.9% of human LTR elements as chimeric-EST-related in the 5 kb human LTR U-zone, but for elements outside the U-zone we observed only 0.9%. All the above results are highly statistically significant (all p-values<2.2e-16; two-sample proportion test), which reinforces the notion that TEs within their U-zones are more likely to be involved in aberrant splicing. It should be pointed out, however, that the splicing events detected by this analysis are of unknown relevance and, indeed, because these TEs are fixed, are unlikely to have significant detrimental effects.

A second prediction is that TEs which were not eliminated from the U-zone would have weaker splicing signals compared with other TEs. To examine this issue, we computationally analyzed potential splice sites within randomly selected solitary LTR sequences in human introns using NNSplice [48] (see Materials and Methods). As shown in Figure 6B, as the distance between the intronic LTR and its nearest exon decreases, the average number and the strength of predicted splice sites in these LTR sequences also decrease. This observation indicates that LTRs carry fewer and weaker cryptic splice sites within the U-zone, especially when they are located in close proximity to exons.

### Abnormal gene splicing linked to polymorphic LTR element insertions near intron boundaries

While the above EST analysis suggests the importance of U-zones in TE-gene interactions, it would be useful to predict which particular intronic TEs are most likely to influence gene transcription based on their size, distance to the nearest exon, orientation, and proximity to particular splice site. To conduct an initial evaluation of this concept, we examined a panel of polymorphic LTR element insertions in inbred mouse strains because they are currently highly active and, as discussed above, their genomic distribution suggests that some are likely detrimental but are maintained due to the artificial breeding environment. In order to take the advantage of the available EST/mRNA data in the B6 reference genome, we restricted our set of intronic polymorphic LTR elements to those present in the B6 mouse strain [45]. After excluding solitary LTRs and complex cases due to multi-gene families, we identified 44 full-length polymorphic LTR elements within the 2 kb U-zone (data not shown). We then inspected each region using the UCSC Genome Browser (mouse genome version: mm9) to look for chimeric ESTs/mRNAs involving the LTR element and the enclosing gene and found such transcripts for 19 of the 44 genes. For most of these 19 genes, the aberrant forms appear to be minor in abundance and it is difficult to estimate their overall impact on gene expression. However, among these 19 genes, transcription of three of them (*Cdk5rap1*, *Adamts13*, and *Wiz*) has been shown to be significantly affected by the embedded LTR element [46], [49], [50].

**Figure 7:**
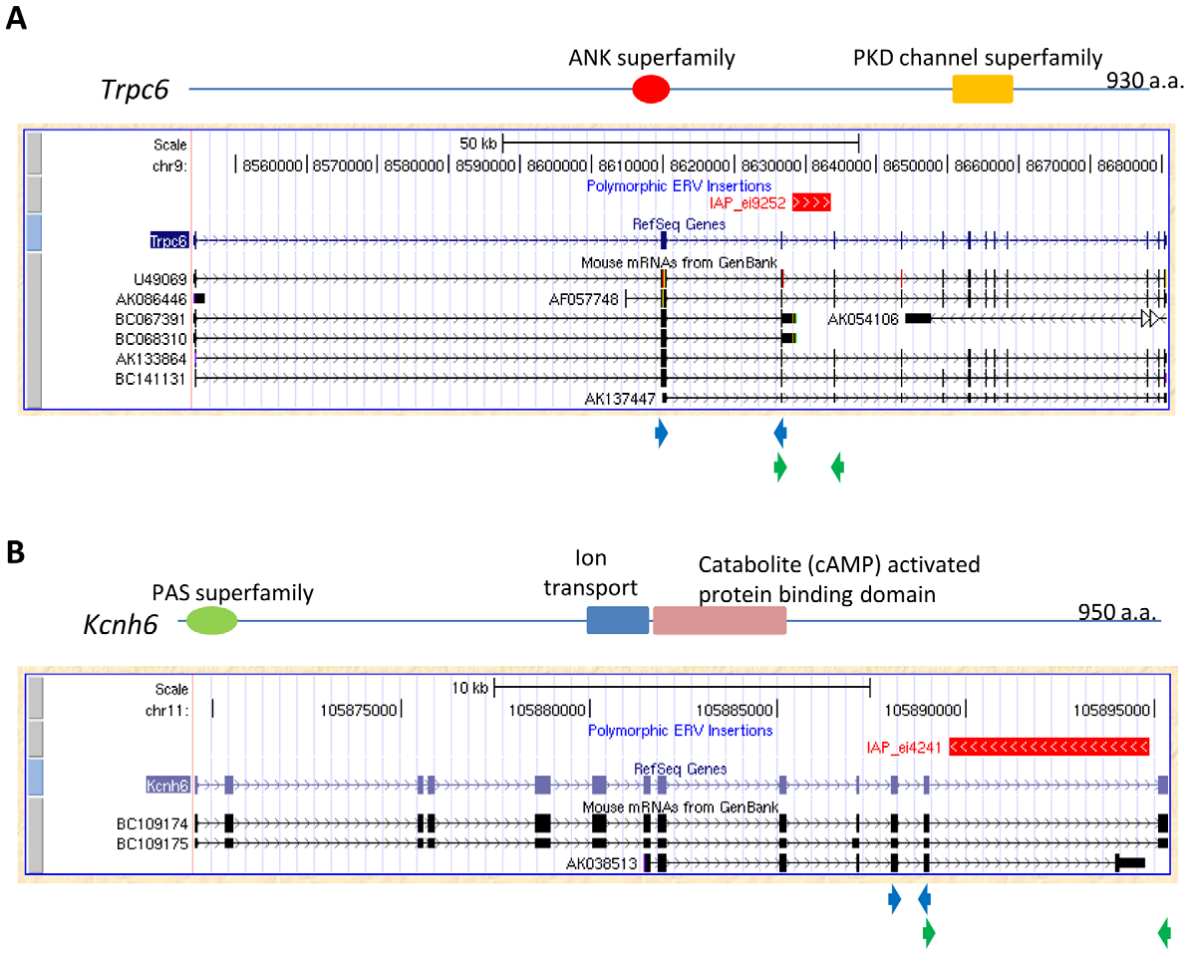
Chimeric transcripts of the *Trpc6* gene and *Kcnh6* gene in mice. Snapshots of the *Trpc6* gene (A) and *Kcnh6* gene (B) in UCSC Genome Browser are shown, with protein domains indicated. The red bar above the RefSeq gene annotation track shows the polymorphic LTR element insertion in B6 mice. For each gene, the mRNA track is shown, including the mRNAs terminating in the LTR element. Positions of primer sets used in the qRT-PCR experiments are indicated as arrowheads below the snapshot for each gene, with the upper pair (blue) for primers upstream of the polymorphic LTR element insertion, and the lower pair (green) for primers flanking the position of the LTR element insertion.

**Figure 8:**
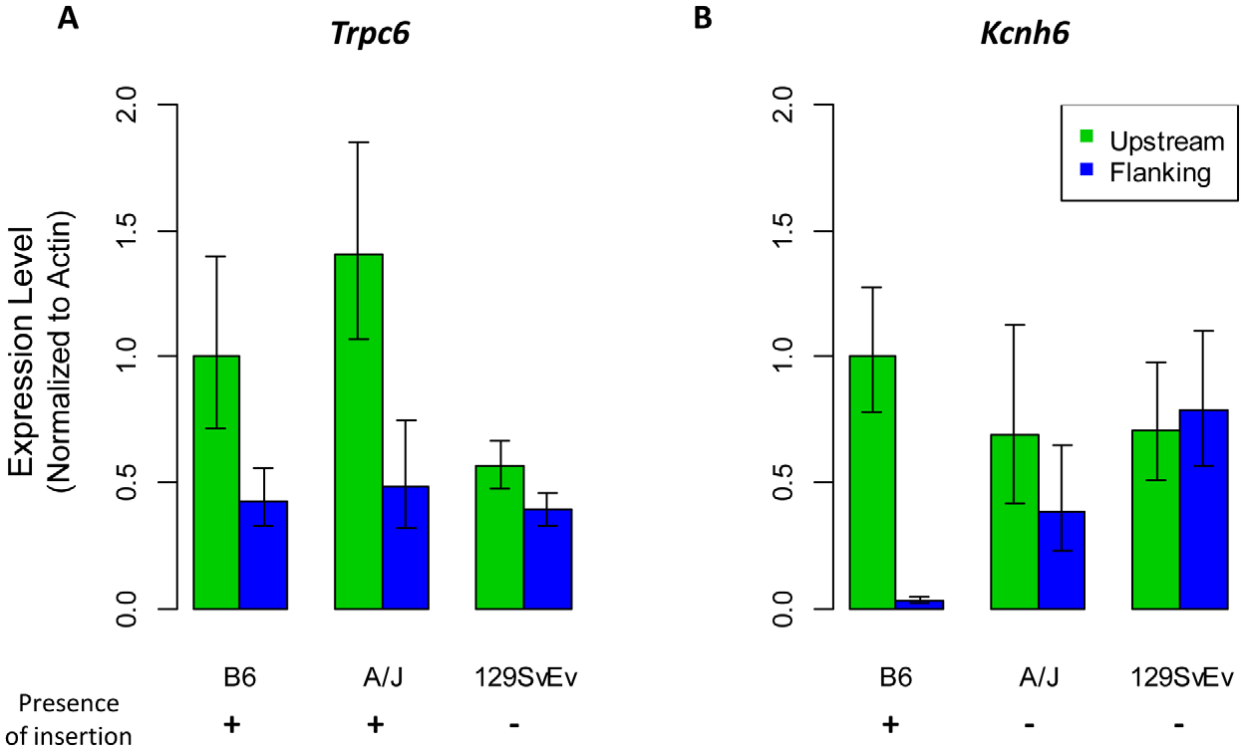
Effect of polymorphic LTR element insertions on transcription of the *Trpc6* and *Kcnh6* genes. Quantitative RT-PCR of the *Trpc6* (A) and *Kcnh6* (B) genes using brain RNA from the indicated mouse strains. Green bars show the amount of transcripts detected by the primer set upstream the polymorphic IAP insertion, and blue bars show the amount of transcripts detected by the primer set flanking the location of the IAP insertion. Each bar represents the mean of at least 4 experiments ± standard deviation, which was first normalized to β-actin levels in the queried strain, and then represented relative to 5′ expression levels for each gene in B6 mice. The plus/minus sign shows the presence/absence of the IAP insertion in the corresponding mouse strain, respectively.

Judging from the frequency of annotated chimeric transcripts, two other genes among the group of 19, *Kcnh6* (potassium voltage-gated channel, subfamily H (eag-related), member 6) and *Trpc6* (transient receptor potential cation channel, subfamily C, member 6), are of special interest. While no evidence of transcriptional disruption caused by LTR element insertions has been reported in the literature for these genes, UCSC Genome Browser snapshots of their deposited mRNAs suggest significant involvement in the transcription of each gene. For *Trpc6*, two of seven mRNAs in the database terminate within a polymorphic IAP LTR element (Figure 7A), and for *Kcnh6*, one of three annotated mRNAs terminates within another IAP insertion (Figure 7B). *Trpc6* plays an important role in vascular and pulmonary smooth muscle cells and its deficiency impairs certain allergic immune responses and smooth muscle contraction [51]. *Kcnh6*, also termed *Erg2* (eag related protein 2), encodes a pore forming (alpha) subunit of potassium channels, and may serve a role in neural activation [52]. To examine the potential effect of the IAP polymorphisms on transcription of these two genes, we first confirmed the presence or absence of these insertions by genomic PCR in a panel of mouse strains including B6, A/J, and 129SvEv. Indeed, an IAP is present in B6 and A/J but not in 129SvEv for the *Trpc6* gene, and the IAP in the *Kcnh6* gene is present only in B6 but not in A/J and 129SvEv (data not shown). Since both genes are highly expressed in the brain, we conducted quantitative RT-PCR on brain cDNA from all three mouse strains by setting one primer pair upstream of the insertion site and another primer pair flanking the insertion site, as indicated in Figure 7. In mouse strains carrying the IAP insertion, we found a significant decrease in the amount of normally spliced transcripts involving exons flanking the ERV insertion, compared with exons upstream of the insertion. In contrast, we saw less difference between the upstream and flanking primer sets in strain(s) without the IAP insertion (Figure 8). The blockage of normal *Kcnh6* transcription is particularly striking, with very little normal splicing occurring for exons flanking the IAP in the B6 strain. These data suggest significant transcriptional interference of these two genes mediated by the embedded IAPs, and it would be interesting to determine if this interference results in phenotypic differences between mouse strains with and without these insertions.

### Conclusions

Over a million TEs have become fixed in human or mouse gene introns during evolution, and the vast majority of them presumably have no functional impact on the gene. Yet, new disease-causing TE insertions do occur in introns and exert detrimental effects mainly by disrupting normal gene transcript processing. The emergence of high throughput technologies has facilitated the discovery of an increasing number of TE germline polymorphisms and somatic insertions in human cancers, with the recent advances on studies of human L1 polymorphisms as the best example [23], [24], [25], [26]. However, little attempt has been made thus far to identify which of these polymorphic or somatically-acquired TEs may contribute to allele-specific gene expression differences and potential phenotypic variation or disease. Methods are therefore needed to evaluate which TEs are most likely to affect gene transcription. Here we have identified intronic underrepresentation zones near exons, where fixed TEs occur less often than expected by chance. Strikingly, all documented human intronic Alu and L1 insertions and most mouse intronic LTR elements known to cause disease are located within these U-zones, strongly suggesting that TE elements in these locations are more likely to cause transcriptional disruptions and be eliminated by selection. Moreover, TEs within their U-zones are more likely to be involved in spliced chimeric transcripts than those located elsewhere in introns, suggesting that some may be slightly detrimental. Presumably in most of these cases the transcriptional effects must be insufficient to cause such insertions to be eliminated by purifying selection. However, it is possible that even apparently subtle effects on gene splicing could have functional consequences. On the other hand, previous studies have also demonstrated that TEs fixed in the host genome can participate in gene transcription, producing alternative transcript isoforms that might have functional importance [12], [53], [54], [55], [56]. Equally important as identifying potentially deleterious TE insertions, it is also of great value to identify fixed TEs that contribute to normal gene expression and cell functionality. The U-zones identified here, coupled with TE size, orientation bias, and location relative to SD or SA sites can all be combined to help predict those TEs with a higher likelihood of functional significance, while yielding new insights into the effects of TEs on gene regulation and evolution.

## Materials and Methods

### Source data

#### TE annotations

The original TE annotation data were obtained from the RepeatMasker tracks of the human hg18 genome and the mouse mm9 genome at the UCSC Genome Browser (http://genome.ucsc.edu) but were further processed to fit this study. Since the annotations of TEs defined by RepeatMasker (http://www.repeatmasker.org) are only fragments based on the similarity to the consensus sequences of different TE families, a single full-length TE element may have multiple RepeatMasker entries if its sequence is not continuous in the genome. Thus, we computationally merged such TE fragments into a single element and counted them only once as an independent TE insertion event in our analyses when they met the following criteria: 1) belong to the same TE family; 2) on the same chromosome; 3) within 10 kb distance; 4) in the same orientation.

#### EST data

The human EST data were also downloaded from the UCSC Genome Browser. Here we used the UCSC Table Browser to download only the spliced EST data, which was stored in the *intronEst* table. According to a reference at the UCSC genome Browser (https://lists.soe.ucsc.edu/pipermail/genome/2008-June/016560.html), TE-only transcripts were not included in these datasets.

### Computer simulation of random TE insertions

To establish a baseline of TE distributions in gene introns, we applied computational simulations of random TE insertions in both the hg18 human genome and the mm9 mouse genome. We used the RefSeq gene annotation data downloaded from the UCSC genome browser (http://genome.ucsc.edu) in our study. For each round of simulation, we generated 1,000,000 random genomic loci across the entire host genome to mimic randomized TE insertions. Next, we divided intronic regions into the following 13 bins with gradually increasing bin size according to their distance to the nearest exon: 0–20 bp, 20–50 bp, 50–100 bp, 100–200 bp, 200–500 bp, 500–1000 bp, 1–2 kb, 2–5 kb, 5–10 kb, 10–20 kb, 20–50 kb, 50–100 kb, >100 kb. The intention of using increasing bin size was to establish a higher resolution at the interesting regions near intron boundaries while also maintain a good overview of other intronic regions. We then calculated the fraction of simulated TE insertions located in each bin with respect to the total simulated insertions in introns. The same simulation process was applied three times and the average was taken for each genome as a control distribution for all further analyses. Finally, our calculation of the “standard error of the mean” based on three rounds of simulations showed a negligible sampling error for each bin (data not shown), confirming the eligibility of using these results to represent the theoretical random TE distribution.

### Normalization of intronic TE distribution by G/C content

To minimize the influence on TE distribution by local G/C content, we corrected our computational simulations of random TE distribution according to the overall G/C preference of each TE class. Specifically, we first performed a genome-wide evaluation of the G/C preference of each TE class by dividing the entire host genome into a set of consecutive 20 kb windows and calculating both the density of each TE class and the G/C density for each window. Then we grouped these 20 kb windows by G/C density level (with a resolution of 1%) and calculated their average TE density at each G/C level. Based on the assumption that TE density should be close to the overall genome-wide TE density anywhere in the genome when there is no G/C preference, we calculated the fold-difference of the actual TE density at each G/C level compared with the genomic background level for each TE class. In this way, we derived a list of ‘fold change’ values of TE density at each G/C level, which was then used as the normalization coefficient to correct the simulated distribution of random TE insertions.

### Calculation of the standardized TE frequency levels

To determine how different the ‘observed’ TE frequency is from the ‘expected’ at each predefined distance bin, we used the concept of *residual* to measure the standardized TE frequency:
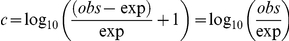
where *c* is the residual of a given distance bin, *obs* is the total observed occurrence of a given TE class in that bin, and *exp* is the expected number of such TE insertions derived from our computational simulations. Common logarithm (log_10_) was used here to equalize the value ranges of over- and under-represented data, and the addition of “1” in the formula is to fulfill the requirement that the subject of logarithm cannot be a negative number. Literally, the absolute value of residual *c* shows the degree of relative difference between the ‘observed’ and ‘expected’, and when *c* is positive, it means the corresponding TE class is overrepresented in this region; when *c* is negative, it means such TE class is underrepresented.

### Computational analysis of potential splice sites in LTRs

Here we used the web-based interface of the NNSplice program (http://www.fruitfly.org/seq_tools/splice.html), which is a bioinformatics tool based on artificial neural networks and used for predicting the presence and the strength of potential splice sites in any given input DNA sequence. Due to the limitation of the maximum length of total input sequences that NNSplice can take, here we only chose sense-oriented solitary LTR sequences (i.e. LTR sequences annotated with a size between 200–600 bp) in human introns in this analysis. The intronic region was divided into a set of consecutive bins with increasing bin size according to the distance from the LTR to the nearest exon as the following: 0–200 bp, 200–500 bp, 500–1000 bp, 1–2 kb, 2–5 kb, >5 kb. For all bins except the first bin, a total number of 100 LTR sequences was sampled randomly for three times independently, and the averaged total numbers and strength of potential splice sites based on the three samples were taken as the final values for each bin. Since the first bin (0–200 bp) contains only 101 cases in total, we took all those cases to calculate the average total number and strength of potential splice sites for this bin without sampling. Notably, when we calculated the average strength of potential splice sites for each bin, only the site with the highest score was considered for each LTR sequence.

### RT-PCR and qRT-PCR of polymorphic LTR insertions in mouse genes

#### DNA and RNA isolation

Primary mouse tissue samples were dissected from healthy adult male C57BL/6J, 129SvEv, and A/J mice, and preserved in RNA later (Ambion). Genomic DNA and total RNA were isolated from the indicated strains and tissues using TRIzol (Invitrogen), according to the manufacturer's specification. Subsequently, nucleic material was quantified using a NanoDrop UV spectrophotometer (Thermo Scientific) and quality was assessed by gel electrophoresis.

#### Confirmation of LTR polymorphisms

The presence/absence of ERV/LTR polymorphisms was initially determined computationally [45]. These predicted events were confirmed by PCR using 50 ng of genomic DNA from C57BL/6J, 129SvEv, and A/J mice. Briefly, primers flanking the predicted LTR polymorphisms in *Kcnh6* (gKcnh6-F: catcccagagctcaaagtgg; gKcnh6-R: tgcaccagtgcatgcatgc) and *Trpc6* (gTrpc6-F: gaagcatgccactctagagc; gTrpc6-R: tgtgcatgattgtgtaggtg) introns were used in a standard Platinum Taq DNA polymerase reaction (94°C-5 min; [94°C-0.5 min; 58°C-0.5 min; 72°C-0.5 min]×35; 72°C-7 min; 4°C-∞).

#### Quantitative RT-PCR

One microgram of total C57BL/6J, 129SvEv, and A/J RNA was reverse transcribed using Superscript III (Invitrogen) following the manufacturer's recommended protocol. The effect of the LTR polymorphisms on the expression of *Trpc6* and *Kcnh6* was assessed by qRT-PCR using primers situated 5′ and 3′ of the respective LTR insertions. Relative quantification of the indicated targets was carried out by the ΔΔC_T_ method, essentially as before [56], using the following primer sets: Kcnh6 5′ (qKcnh6-Ex11-F: cgagagaagctggattgctg; qKcnh6-Ex12-R: ctgtggatgctgaagtagctg); Kcnh6 3′ (qKcnh6-3′-F3: ctcagagttcagagtcgatgc; qKcnh6-3′-R: caccagagatttgtccattgc); Trpc6 5′ (qTrpc6-Ex2-F: cttagccaatgagctggcagtg; qTrpc6-Ex3-R: ccacttcctctgtgtttctgc); Trpc6 3′ (qTrpc6-Ex3-F2: agtatgaagtaaaaaaatttgtggctc; qTrpc6-Ex4-R2: aatggcaacagcaaggaccac); β-actin (β-actin-F: aaggccaaccgtgaaaagat; β-actin-R: gtggtacgaccagaggcatac). Briefly, the amplification efficiency of each primer set was derived across a template dilution series, with the achieved efficiencies as follows: Kcnh6 5′ 87%; Kcnh6 3′ 101%; Trpc6 5′ 98%; Trpc6 3′ 94%; and β-actin 96%. These values were incorporated into calculations used to determine relative expression levels of the target and control genes. Additionally, all *Kcnh6*- and *Trpc6*-specific primer sets were validated to amplify at an ‘equal’ efficiency with respect to β-actin (normalization gene), and thus were determined to be suitable for subsequent ΔΔC_T_ relative quantification qRT-PCR experiments.

## Supporting Information

Text S1 Supporting figure legends. **Figure 1** Intronic distributions of the four major TE classes in human (non-normalized). The distributions of all (A) and full-length (B) intronic TEs in mouse are shown. The x-axis shows a series of intronic regions based on the distance from a TE to the nearest exon. The y-axis represents the standardized frequency of TEs. The red dotted line indicates the expected distribution of TEs based on random computational simulations. Error bars are standard errors derived from the total number of corresponding TEs (sample size) in each bin. **Figure 2** Intronic distributions of the four major TE classes in mouse (normalized). The distributions of all (A) and full-length (B) intronic TEs in mouse are shown. The x-axis shows a series of intronic regions based on the distance from a TE to the nearest exon. The y-axis represents the standardized frequency of TEs and is normalized by G/C content for each TE class. The red dotted line indicates the expected distribution of TEs based on random computational simulations. Error bars are standard errors derived from the total number of corresponding TEs (sample size) in each bin. **Figure 3** Average size of mouse TEs within and outside the U-zone. Each TE class is divided into two groups as shown on the x-axis: one group for elements located within the corresponding U-zone and another group for those beyond. The average size of each TE group is indicated as the horizontal bar within each box, which represents the central 50% of data points of the group. Outliers beyond the 1.5×IQR (interquartile range) whiskers are not shown. P-values shown on top of each boxplot are based on the two sample Wilcoxon test. **Figure 4** Distributional biases of mouse full-length intronic TEs. A) Orientation bias of full-length intronic TEs. The y-axis shows the logarithmic fold-difference of TE frequency between sense and antisense oriented full-length TEs. B) Splice site bias of full-length intronic TEs. The y-axis shows the logarithmic fold-difference of TE frequency between full-length TEs close to the SA site and TEs close to the SD site. The x-axis shows a series of intronic regions based on the distance from a TE to the nearest exon. Error bars are standard errors derived from the total number of corresponding TEs (sample size) in each bin. **Figure 5** Orientation bias of mouse full-length intronic TEs based on proximity to different types of splice sites. Orientation bias of full-length TEs near SD sites (A) and SA sites (B). The x-axis shows a series of intronic regions based on distance of a TE to nearest exon. The y-axis is the logarithmic fold-difference of TE frequency between sense and antisense oriented TEs. Error bars are standard errors derived from the total number of corresponding TEs (sample size) in each bin.
(PDF)

Text S2 Tables of mutagenic TEs in gene introns. **Table 1** Mutagenic human Alu insertions in gene introns. **Table 2** Mutagenic human L1 insertions in gene introns. **Table 3** Mutagenic mouse ERV/LTR insertions in gene introns.
(PDF)
