# Supplementary figures and images for: Distributions of Transposable Elements Reveal Hazardous Zones in Mammalian Introns
Source: PLoS Comput Biol. 2011 May 5;7(5):e1002046. doi: 10.1371/journal.pcbi.1002046 (PMC3088655; doi:10.1371/journal.pcbi.1002046)

Figure 1

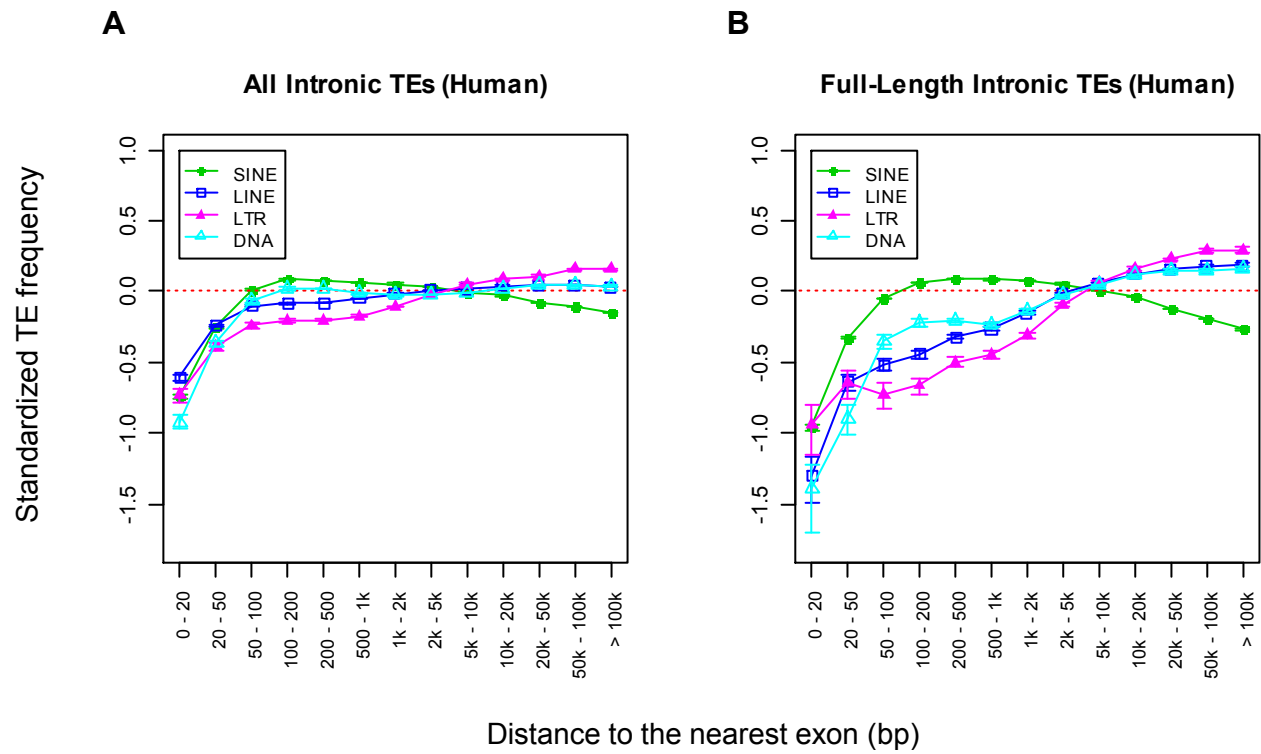

Figure 2

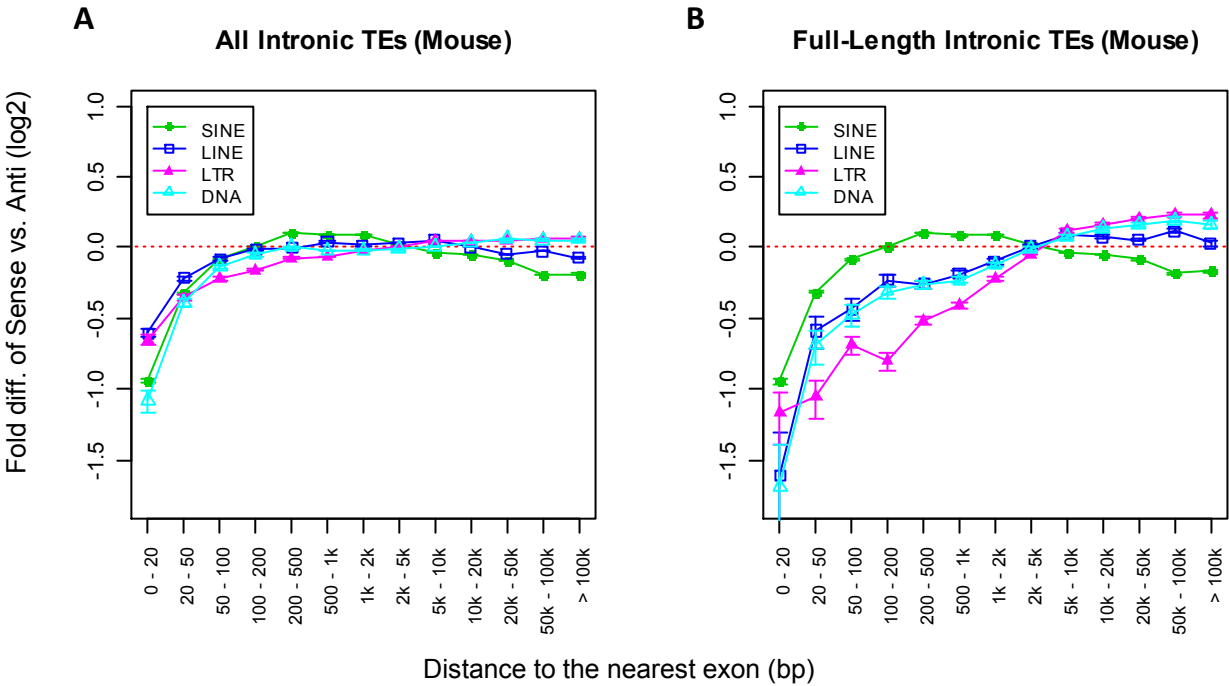

Figure 3

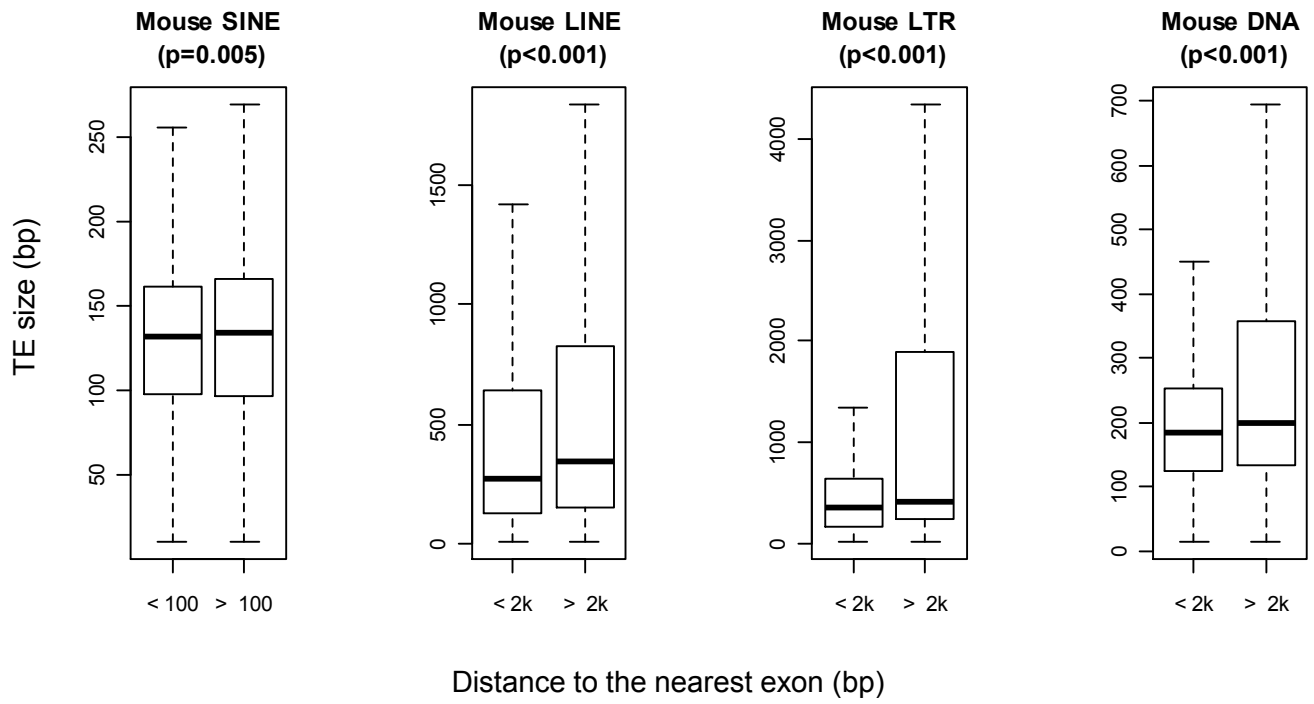

Figure 4

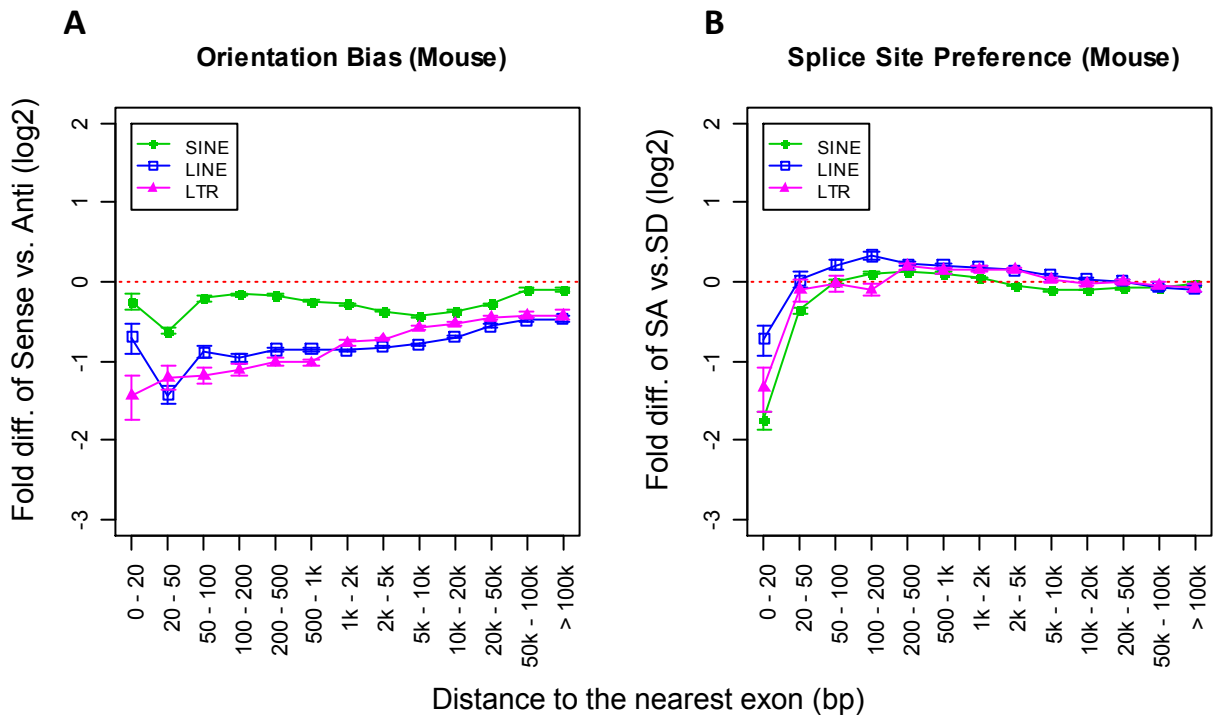

Figure 5

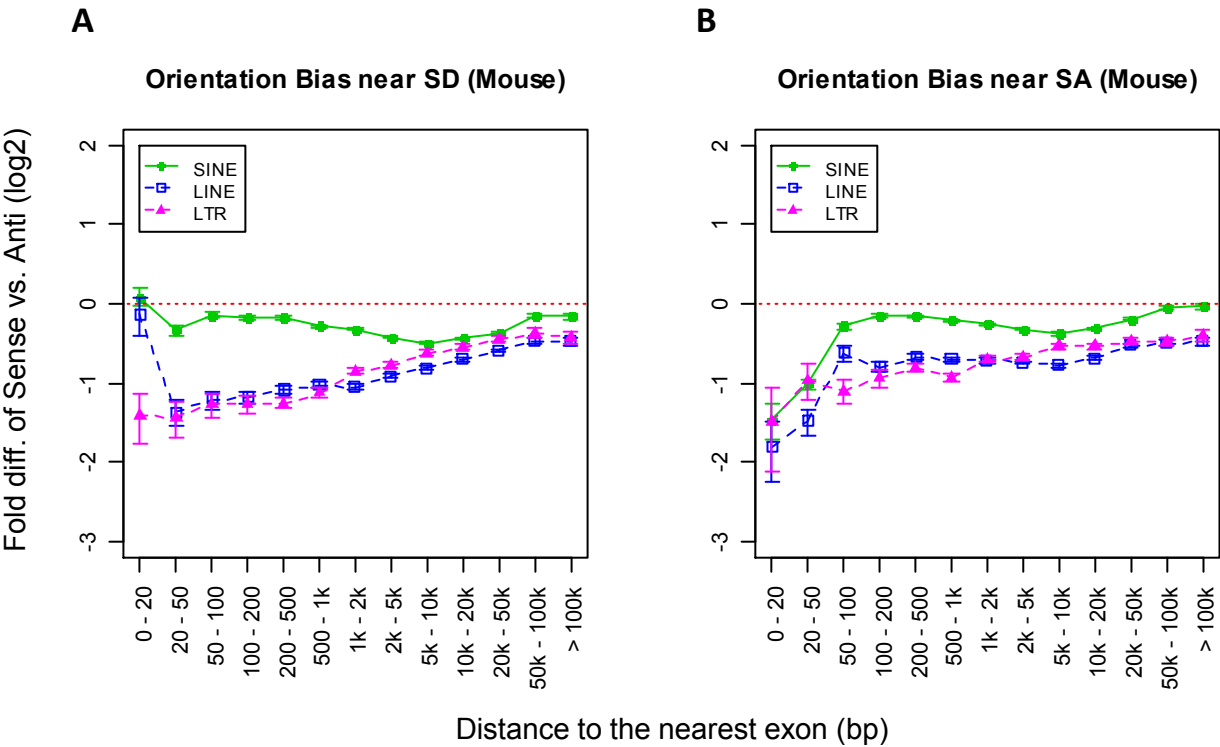

Supplement: Text S1 — Supporting figure legends. Figure 1 Intronic distributions of the four major TE classes in human (non-normalized). The distributions of all (A) and full-length (B) intronic TEs in mouse are shown. The x-axis shows a series of intronic regions based on the distance from a TE to the nearest exon. The y-axis represents the standardized frequency of TEs. The red dotted line indicates the expected distribution of TEs based on random computational simulations. Error bars are standard errors derived from the total number of corresponding TEs (sample size) in each bin. Figure 2 Intronic distributions of the four major TE classes in mouse (normalized). The distributions of all (A) and full-length (B) intronic TEs in mouse are shown. The x-axis shows a series of intronic regions based on the distance from a TE to the nearest exon. The y-axis represents the standardized frequency of TEs and is normalized by G/C content for each TE class. The red dotted line indicates the expected distribution of TEs based on random computational simulations. Error bars are standard errors derived from the total number of corresponding TEs (sample size) in each bin. Figure 3 Average size of mouse TEs within and outside the U-zone. Each TE class is divided into two groups as shown on the x-axis: one group for elements located within the corresponding U-zone and another group for those beyond. The average size of each TE group is indicated as the horizontal bar within each box, which represents the central 50% of data points of the group. Outliers beyond the 1.5×IQR (interquartile range) whiskers are not shown. P-values shown on top of each boxplot are based on the two sample Wilcoxon test. Figure 4 Distributional biases of mouse full-length intronic TEs. A) Orientation bias of full-length intronic TEs. The y-axis shows the logarithmic fold-difference of TE frequency between sense and antisense oriented full-length TEs. B) Splice site bias of full-length intronic TEs. The y-axis shows the logarit [file pcbi.1002046.s001.pdf]
