# Supplementary material for: Distributions of Transposable Elements Reveal Hazardous Zones in Mammalian Introns
Source: PLoS Comput Biol. 2011 May 5;7(5):e1002046. doi: 10.1371/journal.pcbi.1002046 (PMC3088655; doi:10.1371/journal.pcbi.1002046)
Supplement: Text S2 — Tables of mutagenic TEs in gene introns. Table 1 Mutagenic human Alu insertions in gene introns. Table 2 Mutagenic human L1 insertions in gene introns. Table 3 Mutagenic mouse ERV/LTR insertions in gene introns. (PDF) [file pcbi.1002046.s002.pdf]

Text S2: Tables of mutagenic TE insertions in introns

Table 1. Mutagenic intronic Alu insertions in humans

Gene with mutation	Alu subfamily	Orientation#	Nearest splice-site	Distance to exon (bp)	Related diseases	References
FAS	Sb1	-	SA	50	Autoimmune lymphoproliferative syndrome (ALPS)	[1-2]
FGFR2	Yb8	-	SA	10	Apert Syndrome	[3]
APC	Yb9	-	SD	20	Familial Adenomatous Polyposis	[4]
GK	Ya5	-	SA	30	Glycerol kinase deficiency	[5]
F8	Yb9	-	SA	10	Hemophilia A	[1, 6]
OPA1	Yb8	-	SA	21	Autosomal dominant optic atrophy	[7]

The "-" sign represents antisense orientation of the TE with respect to the enclosing gene

Table 2. Mutagenic intronic L1 insertions in humans

Gene with mutation	Orientation	Nearest splice-site	Distance to exon (bp)	Related diseases	References
HBB	+	SA	100	Beta-thalassemia	[8]
FKTN	+	SA	24	Fukuyama-type congenital muscular dystrophy (FCMD)	[9]
RPS7KA3	-	SA	10	Coffin-Lowry syndrome (CLS)	[10]
CYBB	+	SA	265	Chronic granulomatous disease	[1, 11]
RP2	+	SD	653	X-linked retinitis pigmentosa (XLRP)	[12-13]

The "+"/"-" sign represents sense/antisense orientation of the TE with respect to the enclosing gene

Table 3. Mutagenic intronic ERV insertions in mice

Mutation	ERV family	Orientation[#]	Nearest splice-site*	Distance to exon (bp)	References
Ap3d1^{mh2J}	IAP	+	SD	6	[14]
Atrn^{mg}	IAP	-	SD	136	[15]
Atrn^{mg-L}	IAP	+	SD	~420	[15]
Eya1^{bor}	IAP	+	SA	1575	[16]
Gus^{mps2J}	IAP	+	SA	btw. 850-1100	[17]
Lama2^{Pas}	IAP	+	SD	~300	[18]
LamB3^{IAP}	IAP	-	SA	1	[19]
Mgrn1^{md-2J}	IAP	+	SD	616	Ref 20
Mgrn1^{md}	IAP	+	SA	~942	[20-21]
Pitpna^{vb}	IAP	+	SA	~1126	[22]
Spna1^{Dem}	IAP	?	SA	1	[23]
Pofut1^{cax}	IAP	-	SA	24	[24]
Pmca2^{joggle}	IAP	+	SD	~15	[25]
Gria4^{spkw1}	IAP	+	SD	~720	[26]
Zfp69^{SJL}	IAP	?	SA	965	[27]
Adcy1^{brl}	ETn[a]	+	SD	~1700	[28-29]
Cacng2^{stg}	ETn	+	SD	btw. 1500-2100	[30-31]
Cacng2^{stg-3J}	ETn	+	SD	btw. 2500-4100	[30-31]
Clcn1^{adr}	ETn	+	SD	1033	[32]
Fas^{lpr}	ETn	+	SA	3500	[33-35]
Fbxw4^{Dac-2J}	ETn	+	SD	~14000	[36]
Fign^{fi}	ETn	+	SD	~60000	[37]
Foxn1^{nu-Bc}	ETn	-	SA	~5200	[38]
Gli3^{pdn}	ETn	+	SA	24514	[39]
Hk1^{dea}	ETn	?	SA	901	[40]
Lep^{0b-2J}	ETn	+	SD	~3200	[41]
Mip^{Cat-Fr}	ETn	+	SA	~800	[42-43]
Muted^{mu}	ETn	+	SD	2362	[44]
Ttc7^{fsn}	ETn	+	SA	57	[45]
Hsf4^{lop11}	ETn	+	SA	61	[46]
Fig4^{paletremor}	ETn	+	SA	384	[47]
Dysf^{prmd}	ETn	+	SD	495	[48]
Zhx2^{Afr1}	ETn	+	SA	~20600	[49-50]
a	VL30[b]	-	SD	~1200	[51]
Abcb1a^{mds}	MuLV[c]	-	SA	4	[52]
Myo5a^{d}	MuLV	+	SD	~500	[53]
Nox3^{het}	unk[d]	?	SD	~4000	[54]
Pdcd8^{Hq}	MuLV	+	SD	3432	[55]
Pde6b^{rd1}	MuLV	-	SD	1511	[56]
Lmf1^{cld}	MuERV	+	?	<250	[57]

[#] The "+"/"-" indicates orientation of the TE with respect to the enclosing gene; "?" indicates orientation is unknown;
*SD-splice donor sites; SA-splice acceptor sites; "?" indicates that the nearest splice site was not given;
[a]ETn represents the ETn/MusD family; [b]Virus-like 30 element; [c] Murine leukemia virus; [d] “unk" indicates the ERV type was not given.

References for Supporting Tables

1. Chen JM, Stenson PD, Cooper DN, Ferec C: **A systematic analysis of LINE-1 endonuclease-dependent retrotranspositional events causing human genetic disease.** *Hum Genet* 2005, **117:**411-427.
2. Tighe PJ, Stevens SE, Dempsey S, Le Deist F, Rieux-Laucat F, Edgar JD: **Inactivation of the Fas gene by Alu insertion: retrotransposition in an intron causing splicing variation and autoimmune lymphoproliferative syndrome.** *Genes Immun* 2002, **3 Suppl 1:**S66-70.
3. Oldridge M, Zackai EH, McDonald-McGinn DM, Iseki S, Morriss-Kay GM, Twigg SR, Johnson D, Wall SA, Jiang W, Theda C, et al: **De novo alu-element insertions in FGFR2 identify a distinct pathological basis for Apert syndrome.** *Am J Hum Genet* 1999, **64:**446-461.
4. Su LK, Steinbach G, Sawyer JC, Hindi M, Ward PA, Lynch PM: **Genomic rearrangements of the APC tumor-suppressor gene in familial adenomatous polyposis.** *Hum Genet* 2000, **106:**101-107.
5. Zhang Y, Dipple KM, Vilain E, Huang BL, Finlayson G, Therrell BL, Worley K, Deininger P, McCabe ER: **AluY insertion (IVS4-52ins316alu) in the glycerol kinase gene from an individual with benign glycerol kinase deficiency.** *Hum Mutat* 2000, **15:**316-323.
6. Ganguly A, Dunbar T, Chen P, Godmilow L, Ganguly T: **Exon skipping caused by an intronic insertion of a young Alu Yb9 element leads to severe hemophilia A.** *Hum Genet* 2003, **113:**348-352.
7. Gallus GN, Cardaioli E, Rufa A, Da Pozzo P, Bianchi S, D'Eramo C, Collura M, Tumino M, Pavone L, Federico A: **Alu-element insertion in an OPA1 intron sequence associated with autosomal dominant optic atrophy.** *Mol Vis* 2010, **16:**178-183.
8. Kimberland ML, Divoky V, Prchal J, Schwahn U, Berger W, Kazazian HH, Jr.: **Full-length human L1 insertions retain the capacity for high frequency retrotransposition in cultured cells.** *Hum Mol Genet* 1999, **8:**1557-1560.
9. Kondo-Iida E, Kobayashi K, Watanabe M, Sasaki J, Kumagai T, Koide H, Saito K, Osawa M, Nakamura Y, Toda T: **Novel mutations and genotype-phenotype relationships in 107 families with Fukuyama-type congenital muscular dystrophy (FCMD).** *Hum Mol Genet* 1999, **8:**2303-2309.
10. Martinez-Garay I, Ballesta MJ, Oltra S, Orellana C, Palomeque A, Molto MD, Prieto F, Martinez F: **Intronic L1 insertion and F268S, novel mutations in RPS6KA3 (RSK2) causing Coffin-Lowry syndrome.** *Clin Genet* 2003, **64:**491-496.
11. Meischl C, Boer M, Ahlin A, Roos D: **A new exon created by intronic insertion of a rearranged LINE-1 element as the cause of chronic granulomatous disease.** *Eur J Hum Genet* 2000, **8:**697-703.
12. Ostertag EM, Kazazian HH, Jr.: **Biology of mammalian L1 retrotransposons.** *Annu Rev Genet* 2001, **35:**501-538.
13. Schwahn U, Lenzner S, Dong J, Feil S, Hinzmann B, van Duijnhoven G, Kirschner R, Hemberger M, Bergen AA, Rosenberg T, et al: **Positional cloning of the gene for X-linked retinitis pigmentosa 2.** *Nat Genet* 1998, **19:**327-332.
14. Kantheti P, Diaz ME, Peden AE, Seong EE, Dolan DF, Robinson MS, Noebels JL, Burmeister ML: **Genetic and phenotypic analysis of the mouse mutant mh2J, an Ap3d allele caused by IAP element insertion.** *Mamm Genome* 2003, **14:**157-167.
15. Gunn TM, Inui T, Kitada K, Ito S, Wakamatsu K, He L, Bouley DM, Serikawa T, Barsh GS: **Molecular and phenotypic analysis of Attractin mutant mice.** *Genetics* 2001, **158:**1683-1695.
16. Johnson KR, Cook SA, Erway LC, Matthews AN, Sanford LP, Paradies NE, Friedman RA: **Inner ear and kidney anomalies caused by IAP insertion in an intron of the Eya1 gene in a mouse model of BOR syndrome.** *Hum Mol Genet* 1999, **8:**645-653.
17. Gwynn B, Lueders K, Sands MS, Birkenmeier EH: **Intracisternal A-particle element transposition into the murine beta-glucuronidase gene correlates with loss of enzyme activity: a new model for beta-glucuronidase deficiency in the C3H mouse.** *Mol Cell Biol* 1998, **18:**6474-6481.
18. Besse S, Allamand V, Vilquin JT, Li Z, Poirier C, Vignier N, Hori H, Guenet JL, Guicheney P: **Spontaneous muscular dystrophy caused by a retrotransposal insertion in the mouse laminin alpha2 chain gene.** *Neuromuscul Disord* 2003, **13:**216-222.
19. Kuster JE, Guarnieri MH, Ault JG, Flaherty L, Swiatek PJ: **IAP insertion in the murine LamB3 gene results in junctional epidermolysis bullosa.** *Mamm Genome* 1997, **8:**673-681.

20. Phan LK, Lin F, LeDuc CA, Chung WK, Leibel RL: **The mouse mahoganoid coat color mutation disrupts a novel C3HC4 RING domain protein.** *J Clin Invest* 2002, **110:**1449-1459.
21. He L, Lu XY, Jolly AF, Eldridge AG, Watson SJ, Jackson PK, Barsh GS, Gunn TM: **Spongiform degeneration in mahoganoid mutant mice.** *Science* 2003, **299:**710-712.
22. Hamilton BA, Smith DJ, Mueller KL, Kerrebrock AW, Bronson RT, van Berkel V, Daly MJ, Kruglyak L, Reeve MP, Nemhauser JL, et al: **The vibrator mutation causes neurodegeneration via reduced expression of PITP alpha: positional complementation cloning and extragenic suppression.** *Neuron* 1997, **18:**711-722.
23. Wandersee NJ, Roesch AN, Hamblen NR, de Moes J, van der Valk MA, Bronson RT, Gimm JA, Mohandas N, Demant P, Barker JE: **Defective spectrin integrity and neonatal thrombosis in the first mouse model for severe hereditary elliptocytosis.** *Blood* 2001, **97:**543-550.
24. Schuster-Gossler K, Harris B, Johnson KR, Serth J, Gossler A: **Notch signalling in the paraxial mesoderm is most sensitive to reduced Pofut1 levels during early mouse development.** *BMC Dev Biol* 2009, **9:**6.
25. Sun XY, Chen ZY, Hayashi Y, Kanou Y, Takagishi Y, Oda S, Murata Y: **Insertion of an intracisternal A particle retrotransposon element in plasma membrane calcium ATPase 2 gene attenuates its expression and produces an ataxic phenotype in joggle mutant mice.** *Gene* 2008, **411:**94-102.
26. Beyer B, Deleuze C, Letts VA, Mahaffey CL, Boumil RM, Lew TA, Huguenard JR, Frankel WN: **Absence seizures in C3H/HeJ and knockout mice caused by mutation of the AMPA receptor subunit Gria4.** *Hum Mol Genet* 2008, **17:**1738-1749.
27. Scherneck S, Nestler M, Vogel H, Bluher M, Block MD, Berriel Diaz M, Herzig S, Schulz N, Teichert M, Tischer S, et al: **Positional cloning of zinc finger domain transcription factor Zfp69, a candidate gene for obesity-associated diabetes contributed by mouse locus Nidd/SJL.** *PLoS Genet* 2009, **5:**e1000541.
28. Abdel-Majid RM, Leong WL, Schalkwyk LC, Smallman DS, Wong ST, Storm DR, Fine A, Dobson MJ, Guernsey DL, Neumann PE: **Loss of adenylyl cyclase I activity disrupts patterning of mouse somatosensory cortex.** *Nat Genet* 1998, **19:**289-291.
29. Leong WL, Dobson MJ, Logsdon JM, Jr., Abdel-Majid RM, Schalkwyk LC, Guernsey DL, Neumann PE: **ETn insertion in the mouse Adcy1 gene: transcriptional and phylogenetic analyses.** *Mamm Genome* 2000, **11:**97-103.
30. Letts VA, Felix R, Biddlecome GH, Arikkath J, Mahaffey CL, Valenzuela A, Bartlett FS, 2nd, Mori Y, Campbell KP, Frankel WN: **The mouse stargazer gene encodes a neuronal Ca2+-channel gamma subunit.** *Nat Genet* 1998, **19:**340-347.
31. Letts VA, Kang MG, Mahaffey CL, Beyer B, Tenbrink H, Campbell KP, Frankel WN: **Phenotypic heterogeneity in the stargazin allelic series.** *Mamm Genome* 2003, **14:**506-513.
32. Schnulle V, Antropova O, Gronemeier M, Wedemeyer N, Jockusch H, Bartsch JW: **The mouse Clc1/myotonia gene: ETn insertion, a variable AATC repeat, and PCR diagnosis of alleles.** *Mamm Genome* 1997, **8:**718-725.
33. Wu J, Zhou T, He J, Mountz JD: **Autoimmune disease in mice due to integration of an endogenous retrovirus in an apoptosis gene.** *J Exp Med* 1993, **178:**461-468.
34. Adachi M, Watanabe-Fukunaga R, Nagata S: **Aberrant transcription caused by the insertion of an early transposable element in an intron of the Fas antigen gene of lpr mice.** *Proc Natl Acad Sci U S A* 1993, **90:**1756-1760.
35. Chu JL, Drappa J, Parnassa A, Elkon KB: **The defect in Fas mRNA expression in MRL/lpr mice is associated with insertion of the retrotransposon, ETn.** *J Exp Med* 1993, **178:**723-730.
36. Sidow A, Bulotsky MS, Kerrebrock AW, Birren BW, Altshuler D, Jaenisch R, Johnson KR, Lander ES: **A novel member of the F-box/WD40 gene family, encoding dactylin, is disrupted in the mouse dactylaplasia mutant.** *Nat Genet* 1999, **23:**104-107.
37. Cox GA, Mahaffey CL, Nystuen A, Letts VA, Frankel WN: **The mouse fidgetin gene defines a new role for AAA family proteins in mammalian development.** *Nat Genet* 2000, **26:**198-202.
38. Hofmann M, Harris M, Juriloff D, Boehm T: **Spontaneous mutations in SELH/Bc mice due to insertions of early transposons: molecular characterization of null alleles at the nude and albino loci.** *Genomics* 1998, **52:**107-109.
39. Thien H, Ruther U: **The mouse mutation Pdn (Polydactyly Nagoya) is caused by the integration of a retrotransposon into the Gli3 gene.** *Mamm Genome* 1999, **10:**205-209.

40. Peters LL, Lane PW, Andersen SG, Gwynn B, Barker JE, Beutler E: **Downeast anemia (dea), a new mouse model of severe nonspherocytic hemolytic anemia caused by hexokinase (HK(1)) deficiency.** *Blood Cells Mol Dis* 2001, **27:**850-860.
41. Moon BC, Friedman JM: **The molecular basis of the obese mutation in ob2J mice.** *Genomics* 1997, **42:**152-156.
42. Shiels A, Bassnett S: **Mutations in the founder of the MIP gene family underlie cataract development in the mouse.** *Nat Genet* 1996, **12:**212-215.
43. Shiels A, Mackay D, Bassnett S, Al-Ghoul K, Kuszak J: **Disruption of lens fiber cell architecture in mice expressing a chimeric AQP0-LTR protein.** *FASEB J* 2000, **14:**2207-2212.
44. Zhang Q, Li W, Novak EK, Karim A, Mishra VS, Kingsmore SF, Roe BA, Suzuki T, Swank RT: **The gene for the muted (mu) mouse, a model for Hermansky-Pudlak syndrome, defines a novel protein which regulates vesicle trafficking.** *Hum Mol Genet* 2002, **11:**697-706.
45. White RA, McNulty SG, Nsumu NN, Boydston LA, Brewer BP, Shimizu K: **Positional cloning of the Ttc7 gene required for normal iron homeostasis and mutated in hea and fsn anemia mice.** *Genomics* 2005, **85:**330-337.
46. Talamas E, Jackson L, Koeberl M, Jackson T, McElwee JL, Hawes NL, Chang B, Jablonski MM, Sidjanin DJ: **Early transposable element insertion in intron 9 of the Hsf4 gene results in autosomal recessive cataracts in lop11 and ldis1 mice.** *Genomics* 2006, **88:**44-51.
47. Chow CY, Zhang Y, Dowling JJ, Jin N, Adamska M, Shiga K, Szigeti K, Shy ME, Li J, Zhang X, et al: **Mutation of FIG4 causes neurodegeneration in the pale tremor mouse and patients with CMT4J.** *Nature* 2007, **448:**68-72.
48. Ho M, Post CM, Donahue LR, Lidov HG, Bronson RT, Goolsby H, Watkins SC, Cox GA, Brown RH, Jr.: **Disruption of muscle membrane and phenotype divergence in two novel mouse models of dysferlin deficiency.** *Hum Mol Genet* 2004, **13:**1999-2010.
49. Perincheri S, Dingle RW, Peterson ML, Spear BT: **Hereditary persistence of alpha-fetoprotein and H19 expression in liver of BALB/cJ mice is due to a retrovirus insertion in the Zhx2 gene.** *Proc Natl Acad Sci U S A* 2005, **102:**396-401.
50. Perincheri S, Peyton DK, Glenn M, Peterson ML, Spear BT: **Characterization of the ETnII-alpha endogenous retroviral element in the BALB/cJ Zhx2 (Afr1) allele.** *Mamm Genome* 2008, **19:**26-31.
51. Bultman SJ, Klebig ML, Michaud EJ, Sweet HO, Davisson MT, Woychik RP: **Molecular analysis of reverse mutations from nonagouti (a) to black-and-tan (a(t)) and white-bellied agouti (Aw) reveals alternative forms of agouti transcripts.** *Genes Dev* 1994, **8:**481-490.
52. Jun K, Lee SB, Shin HS: **Insertion of a retroviral solo long terminal repeat in mdr-3 locus disrupts mRNA splicing in mice.** *Mamm Genome* 2000, **11:**843-848.
53. Jenkins NA, Copeland NG, Taylor BA, Lee BK: **Dilute (d) coat colour mutation of DBA/2J mice is associated with the site of integration of an ecotropic MuLV genome.** *Nature* 1981, **293:**370-374.
54. Paffenholz R, Bergstrom RA, Pasutto F, Wabnitz P, Munroe RJ, Jagla W, Heinzmann U, Marquardt A, Bareiss A, Laufs J, et al: **Vestibular defects in head-tilt mice result from mutations in Nox3, encoding an NADPH oxidase.** *Genes Dev* 2004, **18:**486-491.
55. Klein JA, Longo-Guess CM, Rossmann MP, Seburn KL, Hurd RE, Frankel WN, Bronson RT, Ackerman SL: **The harlequin mouse mutation downregulates apoptosis-inducing factor.** *Nature* 2002, **419:**367-374.
56. Bowes C, Li T, Frankel WN, Danciger M, Coffin JM, Applebury ML, Farber DB: **Localization of a retroviral element within the rd gene coding for the beta subunit of cGMP phosphodiesterase.** *Proc Natl Acad Sci U S A* 1993, **90:**2955-2959.
57. Peterfy M, Ben-Zeev O, Mao HZ, Weissglas-Volkov D, Aouizerat BE, Pullinger CR, Frost PH, Kane JP, Malloy MJ, Reue K, et al: **Mutations in LMF1 cause combined lipase deficiency and severe hypertriglyceridemia.** *Nat Genet* 2007, **39:**1483-1487.
